# Intranasal Administration of a Monoclonal Neutralizing Antibody Protects Mice against SARS-CoV-2 Infection

**DOI:** 10.3390/v13081498

**Published:** 2021-07-29

**Authors:** Sandro Halwe, Alexandra Kupke, Kanika Vanshylla, Falk Liberta, Henning Gruell, Matthias Zehner, Cornelius Rohde, Verena Krähling, Michelle Gellhorn Serra, Christoph Kreer, Michael Klüver, Lucie Sauerhering, Jörg Schmidt, Zheng Cai, Fei Han, David Young, Guangwei Yang, Marek Widera, Manuel Koch, Anke Werner, Lennart Kämper, Nico Becker, Michael S. Marlow, Markus Eickmann, Sandra Ciesek, Felix Schiele, Florian Klein, Stephan Becker

**Affiliations:** 1 Institute of Virology, Philipps University Marburg, Hans-Meerwein-Straße 2, 35043 Marburg, Germany; halwes@staff.uni-marburg.de (S.H.); kupke@staff.uni-marburg.de (A.K.); rohdecor@staff.uni-marburg.de (C.R.); kraehliv@uni-marburg.de (V.K.); michelle.gellhornserra@uni-marburg.de (M.G.S.); michael.kluever@staff.uni-marburg.de (M.K.); lucie.sauerhering@staff.uni-marburg.de (L.S.); schmidt3@staff.uni-marburg.de (J.S.); wernera4@staff.uni-marburg.de (A.W.); kaemper@staff.uni-marburg.de (L.K.); nico.becker@uni-marburg.de (N.B.); eickmann@staff.uni-marburg.de (M.E.); 2 German Center for Infection Research (DZIF), Partner Site Giessen-Marburg-Langen, 35043 Marburg, Germany; 3 Institute of Virology, Faculty of Medicine and University Hospital Cologne, University of Cologne, 50931 Cologne, Germany; kanika.vanshylla@uk-koeln.de (K.V.); henning.gruell@uk-koeln.de (H.G.); matthias.zehner@uk-koeln.de (M.Z.); christoph.kreer@uk-koeln.de (C.K.); florian.klein@uk-koeln.de (F.K.); 4 Biotherapeutics Discovery, Boehringer Ingelheim Pharma GmbH & Co. KG, Birkendorfer Strasse 65, 88397 Biberach an der Riss, Germany; falk.liberta@boehringer-ingelheim.com (F.L.); felix.schiele@boehringer-ingelheim.com (F.S.); 5 Biotherapeutics Molecule Discovery, Boehringer Ingelheim Pharmaceuticals Inc., Ridgefield, CT 06877, USA; zheng.cai@boehringer-ingelheim.com (Z.C.); fei.han@boehringer-ingelheim.com (F.H.); david_1.young@boehringer-ingelheim.com (D.Y.); guangwei.yang@boehringer-ingelheim.com (G.Y.); michael.marlow@boehringer-ingelheim.com (M.S.M.); 6 Institute for Medical Virology, University Hospital Frankfurt, Goethe University Frankfurt am Main, 60596 Frankfurt am Main, Germany; marek.widera@kgu.de (M.W.); sandra.ciesek@kgu.de (S.C.); 7 Center for Molecular Medicine Cologne (CMMC), University of Cologne, 50931 Cologne, Germany; manuel.koch@uni-koeln.de; 8 Institute for Dental Research and Oral Musculoskeletal Biology and Center for Biochemistry, University of Cologne, 50931 Cologne, Germany; 9 German Center for Infection Research (DZIF), Partner Site Frankfurt am Main, 60596 Frankfurt am Main, Germany; 10 Fraunhofer Institute for Molecular Biology and Applied Ecology (IME), Branch Translational Medicine and Pharmacology, 60596 Frankfurt am Main, Germany; 11 German Center for Infection Research (DZIF), Partner Site Bonn-Cologne, 50931 Cologne, Germany

**Keywords:** SARS-CoV-2, monoclonal antibody, neutralizing antibody, virus, animal experiments, mice, transduction, intranasal administration, topical administration

## Abstract

Despite the recent availability of vaccines against severe acute respiratory syndrome coronavirus type 2 (SARS-CoV-2), there is an urgent need for specific anti-SARS-CoV-2 drugs. Monoclonal neutralizing antibodies are an important drug class in the global fight against the SARS-CoV-2 pandemic due to their ability to convey immediate protection and their potential to be used as both prophylactic and therapeutic drugs. Clinically used neutralizing antibodies against respiratory viruses are currently injected intravenously, which can lead to suboptimal pulmonary bioavailability and thus to a lower effectiveness. Here we describe DZIF-10c, a fully human monoclonal neutralizing antibody that binds the receptor-binding domain of the SARS-CoV-2 spike protein. DZIF-10c displays an exceptionally high neutralizing potency against SARS-CoV-2, retains full activity against the variant of concern (VOC) B.1.1.7 and still neutralizes the VOC B.1.351, although with reduced potency. Importantly, not only systemic but also intranasal application of DZIF-10c abolished the presence of infectious particles in the lungs of SARS-CoV-2 infected mice and mitigated lung pathology when administered prophylactically. Along with a favorable pharmacokinetic profile, these results highlight DZIF-10c as a novel human SARS-CoV-2 neutralizing antibody with high in vitro and in vivo antiviral potency. The successful intranasal application of DZIF-10c paves the way for clinical trials investigating topical delivery of anti-SARS-CoV-2 antibodies.

## 1. Introduction

The pandemic spread of severe acute respiratory syndrome coronavirus type 2 (SARS-CoV-2) poses unprecedented challenges to global public health systems. Facing more than 184 million Coronavirus disease 2019 (COVID-19) cases and 3.9 million fatalities until 7th July 2021 [1], governments worldwide have enacted massive non-pharmaceutical countermeasures to contain the pandemic, with drastic side effects for the global economy and daily life. Although the successful development of several vaccines will reduce the number of new SARS-CoV-2 infections [2,3,4], there is still an urgent need for antiviral interventions to prevent and treat COVID-19. In recent years, monoclonal neutralizing antibodies (nAbs) targeting viral surface proteins have been proven to be effective antiviral interventions against viruses such as the respiratory syncytial virus (RSV), Zaire ebolavirus or human immunodeficiency virus 1 (HIV-1) [5,6,7,8,9]. The spike (S) protein of SARS-CoV-2 has essential functions within the viral replication cycle mediating binding to the cellular receptor human angiotensin-converting enzyme 2 (hACE2) and fusion with the target cell’s endosomal membrane prior to nucleocapsid release into the cytoplasm. The S trimer possesses three receptor-binding domains (RBDs), which dynamically switch between a closed “down” conformation protecting the receptor-binding motif (RBM) and an open “up” state, which recognizes the hACE2 receptor [10,11]. The majority of nABs against SARS-CoV-2 exert their antiviral properties by disrupting the interaction of the RBD with hACE2 and thereby preventing viral entry [12,13]. Depending on their mode of action, RBD-binding nAbs can be categorized into four different classes [13]. While classes 1 and 2 directly block the RBM abolishing receptor binding, classes 3 and 4 bind outside but adjacent to the RBM, which might sterically hinder hACE2 binding. Further, some antibodies only recognize the “up” conformation of the RBD while others specifically bind the “down” conformation or both states. In addition to the RBD-specific nAbs, a smaller number of nAbs were discovered that recognize regions in the N-terminal domain and do not interfere with hACE2 binding [14,15].

Several S-specific nAbs have been described to efficiently neutralize SARS-CoV-2 in vitro and in vivo [16,17,18,19,20,21,22,23]. The efficacy of two S-specific nAbs were already successfully tested in clinical phase III trials and gained emergency use authorization by the US government for the treatment of ambulatory patients with mild to moderate COVID-19 [18,19]. Despite these promising results, all S-specific nAbs available so far have to be administered via intravenous infusion, which is one reason why nAb therapy is cost-intensive and challenging in terms of patient management and compliance. Furthermore, the systemic application of an antibody might be suboptimal with regard to its bioavailability in the lung, the primary site-of-action against respiratory viruses such as SARS-CoV-2 [24]. In order to make nAb therapy more feasible for COVID-19 treatment, it is of great interest not only to identify new potent nAbs but also to investigate alternative approaches for their administration.

Previously, we described the isolation of a large panel of monoclonal SARS-CoV-2 nAbs from twelve SARS-CoV-2-convalescent individuals [25]. One of these nAbs (HbnC3t1p1_F4) showed high neutralizing capacity along with a favorable biochemical profile for large scale production and clinical use. It was previously shown that antibody charge variants can have a substantial impact on stability and pharmacokinetics of an IgG molecule, which is a major challenge for product consistency [26,27]. To reduce this potential cause of heterogeneity, the C-terminal heavy chain lysine of HbnC3t1p1_F4 was removed for further development [27], resulting in a slightly modified antibody named DZIF-10c. We show here by ELISA and surface plasmon resonance (SPR) that DZIF-10c binds the RBD of SARS-CoV-2 S with nanomolar affinity. DZIF-10c efficiently neutralizes both, SARS-CoV-2 pseudoviruses and authentic SARS-CoV-2 with 100% inhibitory concentrations (IC_100_) of 0.01 µg/mL. Furthermore, prophylactic application of DZIF-10c in vivo prevents SARS-CoV-2 infection and mitigates lung pathology in hACE2-transduced mice after both systemic (intraperitoneal, i.p.) and topical (intranasal, i.n.) administration. These results highlight DZIF-10c as a very potent nAb with favorable in vitro characteristics, which efficiently neutralizes infectious SARS-CoV-2 in vivo after i.p. and i.n. administration.

## 2. Materials and Methods

### 2.1. Production of DZIF-10c

Original V-region sequences of the antibody HbnC3t1p1_F4 [25] were formatted unaltered onto the human IgG1 backbone (G1m3 allotype) with the C-terminal lysine removed. Light and heavy chains were configured onto separate pTT5 (licensed from National Research Council of Canada) expression vectors. Briefly, V-regions were codon-optimized for mammalian expression, ordered as vector-overlapping dsDNA fragments from idtdna (Berlin, Germany) and cloned into light and heavy chain pTT5 vectors by infusion reaction methodology (Clontech). The standard transformation procedure was completed utilizing Stellar cells (Clontech) and 500 mL E. coli cultures (LB media with 100 mg/mL carbenicillin) were grown to generate substantial amounts of plasmid DNA for Megaprep plasmid plus purification (Qiagen). Finalized plasmid DNA was sequenced externally (Genewiz) and matched against the reference sequence using Lasergene Seqman software. Following plasmid preparation, all antibodies (DZIF-10c or anti-TNP IgG Isotype control) were expressed in CHO-3E7 (CHO-E) cells using previously described protocols. Briefly, cells were maintained in an actively dividing state in the FreeStyle CHO (FS-CHO) medium before transfection with TransIT Pro (Mirus Bio) following the manufacturer’s recommendations. The transfected culture was maintained for ten days and harvest was done by centrifuging and sterile filtration. Then, cell culture supernatants were loaded onto the MabSelect SuRe column (Cytiva, product number 11003494) pre-equilibrated with Dulbecco’s phosphate buffered saline (DPBS). The columns were then washed with DPBS, DPBS plus 1.0 M NaCl and then DPBS. Then the bound proteins were eluted from the columns with 30 mM sodium acetate (pH 3.5) and the pools was neutralized with 1% volume to a volume of 3 M sodium acetate (pH = 9). The neutralized samples were then sterilely filtered with filtration units, followed by measurements of protein concentration, endotoxin level and purity check by SDS-PAGE and aSEC. The impurities (i.e., aggregates) were then further removed with CEX chromatography by loading the sample onto a prepacked POROS HS50 column (#A36637, Thermo Fisher Scientific, Waltham, MA, USA), washed and then eluted with a salt gradient. The fractions of the eluate were analyzed by aSEC. The high percent monomer fractions were pooled together, and the salt concentration was adjusted to 100 mM NaCl. The proteins were then sterilely filtered and final quality and quantity were assessed (i.e., protein concentration, endotoxin level, and percent monomer by aSEC).

### 2.2. Cryoelectron Microscopy

Fab fragments were generated and purified from full length IgG using a Pierce™ Fab Preparation Kit (Thermo Fisher, Waltham, MS, USA). Purified Fabs were mixed with the SARS-CoV-2 S protein, super stable trimer (AcroBiosystems, Newark, DE, USA) (1.1:1 molar ratio Fab per protomer) to a final protein concentration of 0.2 mg/mL and incubated on ice for 30 min. A total of 3.5 µL of the complex solution were deposited onto a C-flat 1.2/1.3-3C holey carbon copper grid (Electron Microscopy Sciences, Hatfield, PA, USA) that had been freshly glow-discharged for 1 min at 20 mA using a PELCO easiGLOW (Ted Pella). Samples were vitrified in 100% liquid ethane using a Leica EM GP2 automatic plunge freezer (Leica Microsystems, Wetzlar, Germany) after blotting at 10 °C and 85% humidity for 4 s. cryo-EM images were collected on a Titan Krios transmission electron microscope (Thermo Fisher) at 300 kV using a K3 detector (Gatan, Pleasantion, CA USA) in the super-resolution counting mode. Images were energy filtered (20 eV slit) and collected automatically using EPU v. 1.2 software (Thermo Fisher). Each image was composed of 50 individual frames with a total exposure dose of 50 e^-^/Å^2^ and a pixel size of 0.415 Å. Single particle data processing was performed in cryoSPARC v2.15 (Structura Biotechnology Inc., Toronto, ON, Canada) as described below. Super resolution movies were patch motion corrected, Fourier-cropped (factor 1/2) and dose weighted before estimating CTF parameters using the Patch CTF job type. Particles were picked using the reference-free Blob picker, extracted with a box size of 360 pixels (0.83 Å/pixel) and subjected to 2D classification. The best class averages were selected manually (226k particles) to create an ab initio 3D model followed by a homogeneous refinement. Particles were then 3D-classified into four classes and the best resolved classes were selected for a final round of homogenous and subsequent non-uniform refinement (142k particles). For interpretation of the reconstructed 3D cryo-EM map of the Fab-S protein complex PDB 6VSB (S protein) and PDB 7C01 were used (Fab fragment). The Fab fragment coordinates of PDB 7C01 were edited with Sculptor in Phenix (Lawrence Berkeley National Laboratory, Berkeley, CA, USA) to match the sequence of DZIF-10c Fab. Atomic coordinates were rigid body fitted into the 3D cryo-EM map using UCSF Chimera v1.13.1. The resolution of the map did not allow for atomic or per amino acid residue refinement of the coordinates. All figures were created in UCSF Chimera v1.13.1.

### 2.3. Analysis of the Pharmacokinetic Profile of DZIF-10c in NRG and FcRn Mice

NOD.Cg-*Rag1^tm1Mom^ Il2rg^tm1Wjl^*/SzJ (NRG) and B6.Cg-*Fcgrt^tm1Dcr^ Prkdc^scid^* Tg(FCGRT)32Dcr/DcrJ (FcRn) mice (The Jackson Laboratory, Bar Harbor, ME, USA) were bred and maintained at the Decentralized Animal Husbandry Network of the University of Cologne and the experiments were authorized by the State Agency for Nature, Environmental Protection, and Consumer Protection North Rhine-Westphalia (84-02.04.2015.A353). To determine in vivo pharmacokinetic profiles of individual antibodies in NRG and FcRn mice, longitudinal serum samples were collected after a single intravenous injection of 0.5 mg of the monoclonal antibody in PBS. Serum samples were stored at −20 °C until analysis and a sample obtained from each mouse before the start of the experiment was used to confirm the baseline absence of human IgG. Human IgG serum concentrations were determined as described previously with minor modifications [28]. High-binding ELISA plates (Corning) were coated with goat anti-human IgG (Jackson ImmunoResearch, West Grove, PA, USA) at a concentration of 2.5 µg/mL for 10 h at room temperature (RT, NRG mice) or 2 h at 37 °C (FcRn mice), followed by blocking with a blocking buffer (2% BSA (Carl Roth), 1 µM EDTA (Thermo Fisher) and 0.1% Tween-20 (Carl Roth) in PBS) for 80 min at 37 °C (NRG mice) or 120 min at RT (FcRn mice). Subsequently, a human IgG1 kappa standard purified from myeloma plasma (in duplicates per plate, Sigma-Aldrich) and serum samples (starting at a 1:20 dilution) were incubated in serial dilutions in PBS for 75–90 min at room temperature (RT). For detection, HRP-conjugated goat anti-human IgG (Jackson ImmunoResearch) diluted 1:1000 in the blocking buffer was applied for 75–120 min at RT. Finally, the optical density at 415 nm was determined using a microplate reader (Tecan) after the addition of ABTS (Thermo Fisher). Between each step, plates were washed with 0.05% Tween-20 in PBS. Human serum IgG concentrations were calculated using the plate-specific IgG1 standard curve.

### 2.4. Analysis of DZIF-10c In Vivo Efficacy after the SARS-CoV-2 Challenge

All challenge animal experiments were performed in accordance with the German animal protection laws and were authorized by the regional authorities (RP Gießen, G44/2020). The hACE2 transduction mouse model for SARS-CoV-2 and associated procedures were designed based on a previously established model for MERS-CoV [29,30,31] and were described recently [32]. Briefly, 6–8 week old BALB/c mice were purchased from Charles River Laboratories and housed under specific pathogen-free conditions in isocages in the animal facility at the Institute of Virology Marburg. Prior to the viral challenge, all mice were inoculated intratracheally with 5 × 10^8^ PFU Ad_ACE2-mCherry (ViraQuest Inc., North Liberty, IA, USA) in order to induce a pulmonary expression of hACE2. To this end, mice were anesthetized by ketamine/xylazine and intubated using an intravenous catheter (22 G, 0.9 mm × 25 mm, Braun, Germany). The adenovirus was diluted in DMEM and 50 µL of the dilution were applied through the catheter (2 × 25 µL with a short interruption). Anesthesia was partially antagonized by atipamezole to avoid unnecessary stress to the animals. Three days post transduction, mice were inoculated via the intranasal route with 1.5 × 10^4^ TCID50 SARS-CoV-2 (BavPat1/2020 isolate, European Virus Archive Global # 026V-03883) in the BSL-4 facility (Institute of Virology, Philipps University Marburg, Germany) under brief isoflurane anesthesia. Using a standard pipette, the virus solution was applied in small drops alternately on each nostril and was actively inhaled by the mice, which were held in an upright, slightly supine position. A total volume of 50 µL was applied in two steps—first 30 µL and, after a short interruption, an additional 20 µL. Animals were monitored daily and clinical scores including body weight changes were documented. On day four post infection mice were sacrificed and lung samples were collected. Lung samples were taken from the upper left lung lobe and were homogenized in 1 mL DMEM with ceramic and glass beads (Lysing Matrix H 500, 2 mL tube, MP Biomedicals) in a mixer mill (Schwingmühle MM 400, Retsch, Haan, Germany) for 5 min at 30 Hz. To remove tissue debris, homogenates were centrifuged for 5 min at 2400 rpm. A total of 40 µL of fresh homogenates were used for a TCID_50_ assay. A further 100 µL of the homogenate were used for RNA isolation.

The application of monoclonal antibodies (DZIF-10c or IgG isotype control) was performed at a dose of 40 mg/KG under short isoflurane anesthesia. Antibodies were administered systemically via the intraperitoneal route using a 25G needle. In the case of topical administration, mouse lungs were instilled via the intranasal route as it was described for the infection (30 µL at maximum, depending on animal weights). Antibody treatment was conducted on day one before infection (prophylactic regimen) or twice on day one and three after infection (therapeutic regimen). In order to ensure a safe administration of antibodies, mice were anesthetized shortly with isoflurane before each treatment.

### 2.5. Histopathological Examination of Lung Tissue

Lungs were collected on day four post challenge with SARS-CoV-2 and processed for histological analysis as described before [29]. Tissue was fixed in formalin and embedded in paraffin. For histopathological analysis, sections were cut with a Leica RM2255 microtome (Leica Biosystems) and stained with hematoxylin and eosin (H&E). To investigate the presence of viral RNA, lungs were mounted on slides and analyzed via in-situ hybridization. To this end, the RNAscope^®^ 2.5 HD Assay—RED Kit from Bio-Techne (Cat. No. 322360) was used according to the manufacturer’s instructions. Briefly, mounted slides were baked at 60 °C, deparaffinized with xylene and 100% ethanol and pretreated with RNAscope^®^ Pretreatment Reagents (Cat. No. 322300 and 322000) to enable access to the target RNA. Subsequently, an RNA-specific probe, targeted against the S gene of the SARS-CoV-2 (Cat. No. 848561), was hybridized to the RNA. The Fast Red substrate was administered to the samples, allowing signal detection. The slides were counterstained with Gill’s Hematoxylin I and 0.02% ammonia water. A RNAscope^®^ Negative Control Probe (Cat. No. 310043) was used in parallel to monitor background staining.

### 2.6. Statistical Analysis

In order to test for statistical differences in viral load analyses (RT-qPCR and TCID_50_ assays) and histopathological scores between treated and non-treated animals, a nonparametric analysis using the Mann–Whitney test was performed. Results were declared significant at *p* < 0.05. All statistical analyses were performed using GraphPad Prism.

## 3. Results

### 3.1. DZIF-10c Displays an Extraordinary Neutralizing Capacity against SARS-CoV-2 and Remains Active against SARS-CoV-2 Variants B.1.1.7 and B.1.351

**Figure 1:**
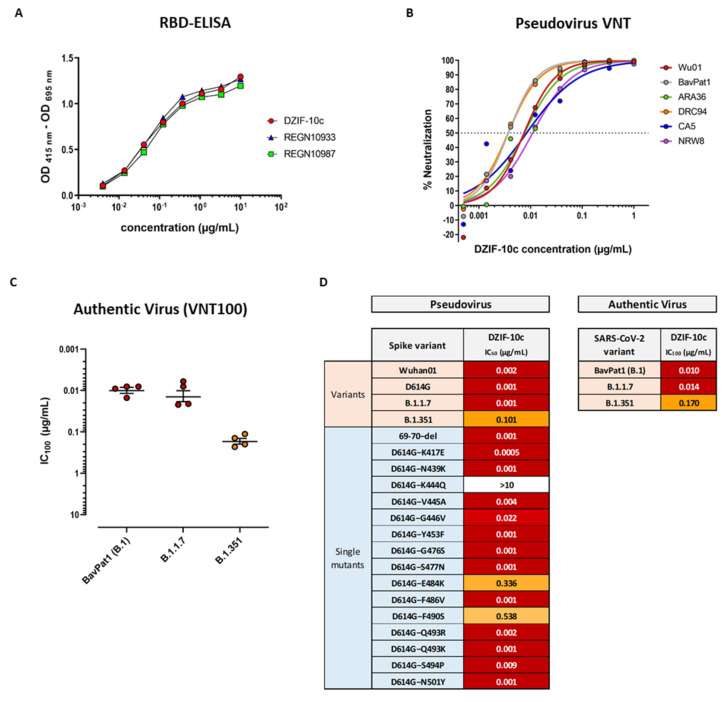
DZIF-10c binds and neutralizes SARS-CoV-2 with high potency. (**A**) Interaction of DZIF-10c, REGN10933 and REGN10987 with SARS-CoV-2 S RBD measured by ELISA. (**B**) Neutralizing activity of DZIF-10c against SARS-CoV-2 pseudoviruses bearing S proteins from different circulating strains. The dotted line indicates 50% neutralization (IC_50_). (**C**) Neutralization of authentic SARS-CoV-2 (BavPat1, B.1) and SARS-CoV-2 variants B.1.1.7 and B.1.351 by DZIF-10c measured by VNT100. Neutralization was defined as the complete inhibition of CPE (IC_100_). Circles represent geometric means from four independent experiments. Lines and error bars indicate the overall mean with SEM. (**D**) Summary of the neutralizing activity (IC_50_ or IC_100_) of DZIF-10c against SARS-CoV-2 pseudoviruses bearing various mutations in the S protein and against authentic SARS-CoV-2 variants.

To compare the characteristics of DZIF-10c with other antibodies that have already shown clinical efficacy in phase 3 studies [18], we analyzed the binding of DZIF-10c and REGN10933 and REGN10987 to the RBD of SARS-CoV-2 S (Figure 1A). All three antibodies showed specific binding to the RBD of SARS-CoV-2 S by ELISA with half-maximal effective concentrations (EC_50_) between 0.046 µg/mL (DZIF-10), 0.057 µg/mL (REGN10933) and 0.061 µg/mL (REGN10987). S-binding was further confirmed by ELISA against the full trimeric S ectodomain, a truncated N-terminal S1 subunit and a monomeric S ectodomain while no binding to the unrelated Zaire ebolavirus glycoprotein was observed (Figure S1). Using SPR we could further show that DZIF-10c targets the RBD of SARS-CoV-2 S with high affinity indicated by an equilibrium dissociation constant (K_D_) of 1.09 ± 0.22 nM (Figure S2). As these results highlight DZIF-10c as a very potent S-binding antibody, we further characterized its neutralizing properties. The in vitro neutralizing activity of DZIF-10c was first evaluated against a panel of six pseudovirus variants [33,34]. DZIF-10c demonstrated potent neutralizing activity against all SARS-CoV-2 S variants tested, including a variant carrying the D614G mutation, with an average IC_50_ of 0.007 µg/mL (Figure 1B). In line with these results, DZIF-10c efficiently neutralized authentic SARS-CoV-2 (BavPat1, lineage B.1) with a mean IC_100_ of 0.01 µg/mL (Figure 1C,D). Since the emergence of several SARS-CoV-2 variants of concern (VOC) showed that certain amino acid changes (e.g., E484K and N501Y) in the RBD of the S protein may completely abolish the neutralization of antibodies raised against the Wuhan strain of SARS-CoV-2 [35,36,37,38,39], we further tested the ability of DZIF-10c to neutralize pseudovirus particles bearing S proteins with single point mutations in the Wuhan background, the 69–70 deletion mutant and authentic VOCs.

The pseudovirus neutralization assay revealed that the neutralization capacity of DZIF-10c was not affected by 16 out of 19 tested point mutations in the RBD. Although DZIF-10c was able to efficiently neutralize pseudovirus particles bearing the B.1.1.7 spike variant, reduced neutralizing activity was observed against the E484K and F490S mutants and the B.1.351 variant as demonstrated by an about 2.5-log-fold reduction in the IC_50_ (Figure S3 and Figure 1D). Importantly, activity of DZIF-10c was unchanged against the currently circulating authentic VOC B.1.1.7 and, though reduced by 17-fold, also active against VOC B.1.351 (Figure 1C,D).

### 3.2. Structural Analysis Indicates Binding of DZIF-10c to the Prefusion Conformation of S Adjacent to the Receptor Binding Motif

**Figure 2:**
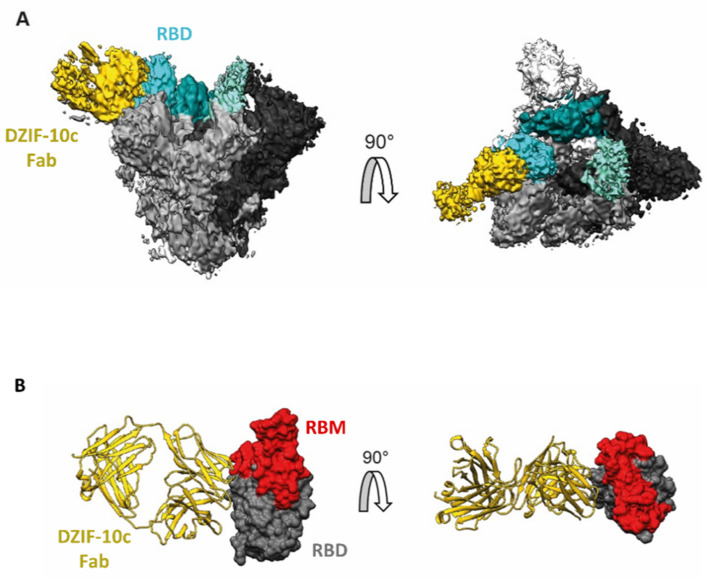
Cryo-EM map of the DZIF-10c Fab/SARS-CoV-2 S complex. (**A**) Cryo-EM map with S protein colored in gray shades (according to the three protomers), density corresponding to the RBDs colored in cyan shades and density corresponding to the Fab fragment colored in yellow. left: side view; right: top view. (**B**) Approximate binding position and angle of DZIF-10c relative to the RBD. DZIF-10c colored in yellow, RBD (PDB-6XDG) colored in grey and receptor binding motif (RBM) on RBD colored in red.

To investigate the structural basis of the neutralizing activity of DZIF-10c, the protein structure of the DZIF-10c Fab/SARS-CoV-2 S complex was determined using cryoelectron microscopy (cryo-EM) at a global resolution of 3.7 Å according to the 0.143 criteria [40] (Figure S4B,C). The reconstructed 3D cryo-EM map of the analyzed Fab-antigen complex clearly shows the trimeric shape of the S protein and additional density for a single DZIF-10c Fab, bound to one of the three S protein protomers (Figure 2A). The reconstructed S protein is arranged in a conformation showing one RBD in the “up”-conformation, while the remaining two RBDs reside in the “down”-conformation corresponding to the prefusion conformation [10].

The DZIF-10c Fab fragment binds to the RBD in “up”-conformation and is arranged nearly perpendicular, relative to the symmetry axis of the S protein trimer (Figure 2A). No Fab density was observed close to the remaining two RBDs in the “down”-conformation, despite the 3:1 molar excess of Fab to the S protein trimer suggesting that DZIF-10c preferably binds to the “up”-conformation of the RBD, which corresponds to the activated prefusion state of the S protein. Furthermore, the data show that the approximated binding position and angle of DZIF-10c did not or only peripherally interfered with the ACE2 binding motif on the RBD (Figure 2B), pointing to a mode of inhibition independent from directly blocking the ACE2 binding.

### 3.3. DZIF-10c Shows a Favorable Pharmacokinetic Profile In Vivo

**Figure 3:**
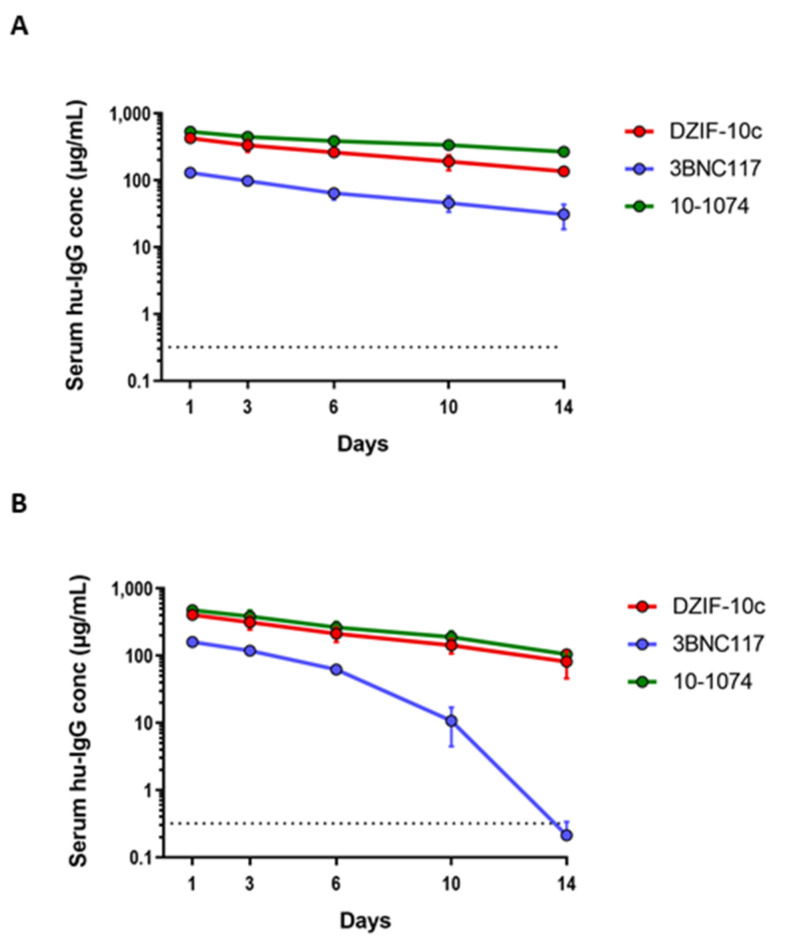
DZIF-10c shows a favorable pharmacokinetic profile in huFcRn and NRG mice. (**A**) Pharmacokinetic profile of DZIF-10c and two human anti-HIV-1 IgG1 antibodies in mice expressing the human neonatal Fc receptor after i.v. injection of a 0.5 mg dose. (**B**) The pharmacokinetic profile of DZIF-10c and two human anti-HIV-1 IgG1 antibodies in NRG mice after i.v. injection of a 0.5 mg dose. Antibody levels were determined by human IgG-specific ELISA. N = 4 per group and antibody. Symbols show means, error bars indicate standard deviation.

In view of its promising in vitro properties, we next characterized the pharmacokinetic profile of DZIF-10c after a single i.v. injection in two different mouse models. First, DZIF-10c was investigated in severe combined immunodeficient (SCID) mice expressing a human alpha chain Fc molecule instead of the endogenous mouse Fcgrt (Figure 3A). The human neonatal Fc receptor (huFcRn) reduces lysosomal degradation of human IgG and plays a key role in antibody half-life. Mice genetically engineered to express the human neonatal Fc receptor are used as a surrogate model for antibody pharmacokinetics in humans [41]. After administration of an antibody dose of 0.5 mg in PBS, antibody serum levels were determined by a human IgG ELISA using purified human myeloma IgG as the on-plate standard. In these mice, DZIF-10c showed a favorable pharmacokinetic profile similar to two HIV-1-neutralizing human IgG antibodies that are currently in clinical investigation and show an in vivo half-life of approximately 2 (3BNC117) to 3 weeks (10-1074) in humans [7,28,42]. In addition, DZIF-10c was administered to immunodeficient NRG mice that do not express the IL-2 receptor common gamma chain, carry a knock-out mutation in the *Rag1* gene and do not develop murine lymphocytes or NK cells (Figure 3B). This model has previously been used to faithfully reproduce the overall pharmacokinetic characteristics of antiviral antibodies in humans [6,7,42,43]. Again, compared to the reference antibodies the pharmacokinetic profile of DZIF-10c was similar or prolonged. Neither mouse model showed accelerated clearance and/or serum elimination of DZIF-10c.

### 3.4. DZIF-10c Efficiently Protects hACE2-Transduced Mice from Infection with SARS-CoV-2

To demonstrate the antiviral efficacy in vivo, we analyzed the prophylactic and therapeutic potential of DZIF-10c after the SARS-CoV-2 challenge in a hACE2 transduction mouse model. Since mice are naturally not susceptible to SARS-CoV-2 infection due to incompatibility of the S protein to murine ACE2 [44,45,46], we transduced BALB/c mice intratracheally with an adenoviral vector carrying the genetic information for hACE2 and the reporter mCherry (Ad_ACE2-mCherry). This approach is based on a previously published model for middle east respiratory syndrome coronavirus (MERS-CoV) [29,30] and has been shown to be suitable for modeling SARS-CoV-2 infection and preclinical testing of a SARS-CoV-2 vaccine candidate [32]. Moreover, this model was used successfully by several other groups to study SARS-CoV-2 pathogenesis and antiviral interventions [22,47,48]. The recombinant adenovirus additionally encodes the fluorescent protein mCherry, which allows the assessment of the transduction efficiency (Figure S6). 

**Figure 4:**
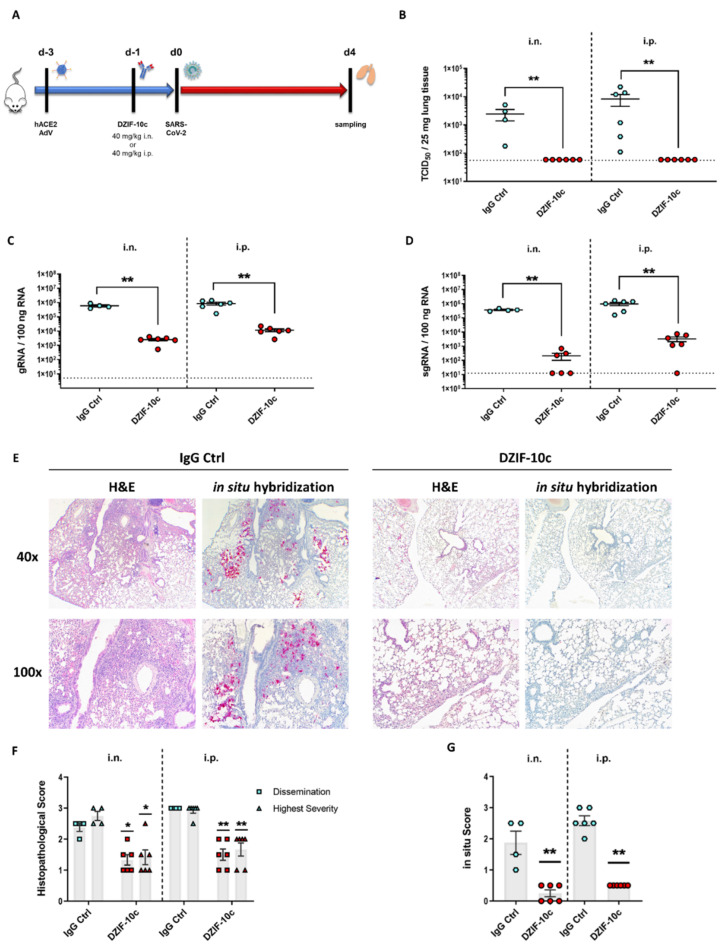
Viral load and histological analysis of hACE2-transduced BALB/c mice prophylactically treated with DZIF-10c. (**A**) Study plan for the prophylactic dose regimen. BALB/c mice were transduced with Ad_ACE2-mCherry three days before infection and treated i.n. or i.p. with 40 mg/KG of body weight DZIF-10c or an IgG control antibody one day prior to challenge with SARS-CoV-2. On day four post infection, the animals were euthanized, and samples were collected. Two mice from the i.n. control group had to be excluded from the analysis due to insufficient transduction efficiency. (**B**) Infectious SARS-CoV-2 titer in lung homogenates on day four post infection determined by the TCID_50_ assay. (**C**,**D**) SARS-CoV-2 gRNA and sgRNA in lung homogenates on day four post infection determined by RT-qPCR. Error bars represent mean ± SEM. Statistical analyses were performed using Graph Pad Prism and the Mann–Whitney test. *: *p* ≤ 0.05; **: *p* ≤ 0.01. Dotted lines indicate the lower limit of detection. (**E**) Histopathological analysis of the lungs by H&E staining and in situ hybridization of viral RNA. Images were acquired at a magnification of 40× or 100×. (**F**,**G**) Quantification of histopathological scores for dissemination and highest severity of inflammation and dissemination of viral RNA. Error bars represent mean ± SEM. Statistical analyses were performed using Graph Pad Prism and the Mann–Whitney test. *: *p* ≤ 0.05; **: *p* ≤ 0.01.

First, we assessed if DZIF-10c can protect hACE2-transduced mice from SARS-CoV-2 infection in a prophylactic setting. To this end, we transduced BALB/c mice with Ad_ACE2-mCherry on day three prior to the challenge followed by a single dose of 40 mg/KG body weight DZIF-10c or an IgG isotype control antibody on day one prior to the challenge (Figure 4A). The antibodies were administered by two different routes to model a systemic (i.p.) or a topical (i.n.) administration. The i.n. application was supposed to mimic a pulmonary delivery via the nasal and respiratory tract. One day after treatment, mice were challenged with SARS-CoV-2 and monitored daily for changes in body weight, behavior and appearance. Four days post challenge, the mice were euthanized, and the lungs were collected for viral load determination and histological analyses. Neither significant body weight changes nor clinical symptoms were observed in any of the groups (Figure S7), which is in line with previous reports of hACE2- transduced mice [32,49]. Importantly, this result indicated that the presence of DZIF-10c did not induce antibody-dependent enhancement (ADE). Regarding viral load, we observed high titers of infectious SARS-CoV-2 in lung homogenates of all mice that received the IgG isotype control antibody with 2.46 × 10^3^ (±1.06 × 10^3^) TCID_50_/25 mg tissue in the i.n. group and 8.35 × 10^3^ (±3.76 × 10^3^) TCID_50_/25 mg tissue in the i.p. group (Figure 4B). In contrast, no infectious virus was detected in mice prophylactically treated with DZIF-10c independent on the route of delivery, indicating efficient neutralization of SARS-CoV-2 in vivo. In line with these results, viral genomic RNA (gRNA) measured by RT-qPCR was reduced significantly by approximately two log scales in DZIF-10c-treated mice (Figure 4C). Intriguingly, in animals receiving the antibody i.n., the decrease was approximately three times greater compared to animals of the i.p. group. Due to the possibility that gRNA measured by RT-qPCR is partially derived either from input virus or intact, but neutralized virions that are not yet cleared by the immune system, we further analyzed subgenomic RNA (sgRNA) levels to assess active SARS-CoV-2 replication. Confirming the absence of infectious particles in the TCID_50_ assay, sgRNA levels dropped dramatically after prophylactic DZIF-10c treatment by more than three log scales in the i.n. and more than two log scales in the i.p. group (Figure 4D). As expected, sgRNA was detected in high amounts in all control mice, which indicates active replication that is prevented in the case of prophylactic treatment with DZIF-10c. Again, we could observe a 6-fold stronger reduction of sgRNA in animals treated via the i.n. route compared to i.p. application. 

Next, we evaluated the impact of DZIF-10c on lung histopathology after hematoxylin–eosin (H&E) staining. In lungs of control mice, we observed interstitial pneumonia with multiple, partially confluent foci and distinct lymphohistiocytic infiltrations in the interstitium and in the perivascular space (Figure 4E). Importantly, prophylactic treatment with DZIF-10c markedly mitigated lung pathology with all animals showing only mild signs of inflammation.

To quantify the histopathological findings, the overall dissemination of inflammation in the lung and the highest severity in terms of immune cell infiltration and damage to the lung architecture was assessed using a scoring system from 0 (no inflammation) to 4 (most severe) (Figure S9). Confirming the visual observation, histopathological scores were reduced 2-fold in DZIF-10c treated mice (Figure 4F). To compare the amounts of SARS-CoV-2 RNA, especially in inflamed areas, we further visualized viral RNA using in situ hybridization with SARS-CoV-2-specific probes. Consistent with our previous results, we detected high amounts of viral RNA in samples from control animals that particularly concentrated in regions with inflammation (Figure 4E,G) whereas in DZIF-10c-treated mice, viral RNA was only detectable in single cells without spreading to the surrounding tissue. This difference was more pronounced in i.n. treated mice as viral RNA was completely absent in three animals in this group. Altogether, these findings indicate that prophylactic treatment with DZIF-10c, either administered i.p. or i.n., efficiently protected hACE2-transduced mice from infection with SARS-CoV-2 and SARS-CoV-2-related lung pathology. Comparing the different routes of application, the i.n. administration of DZIF-10c seemed to be more effective than systemic application. 

**Figure 5:**
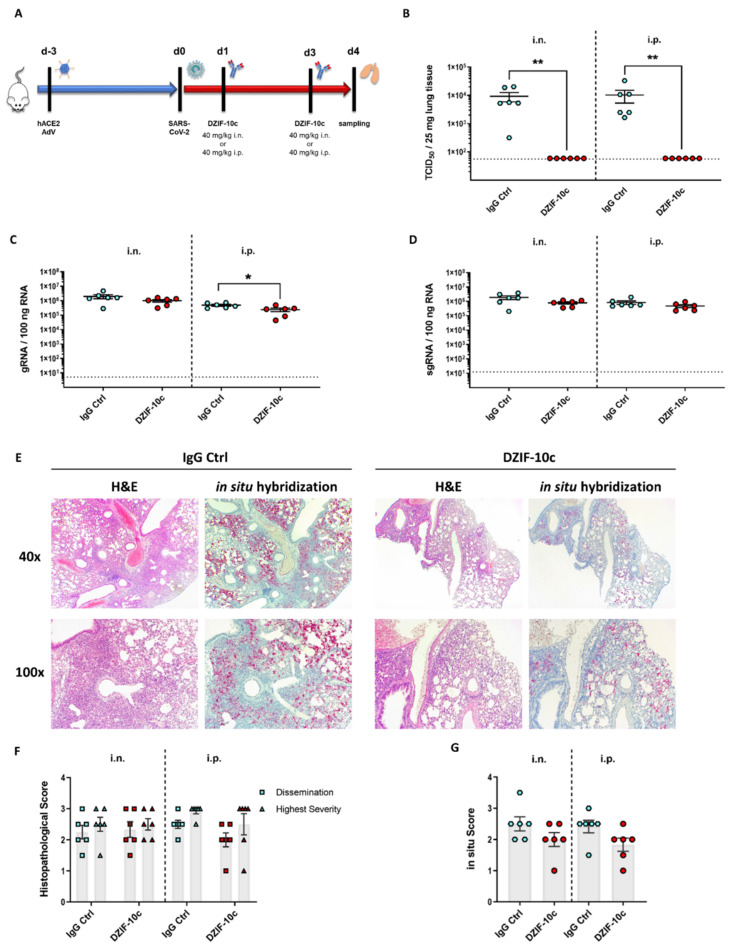
Viral load and histological analysis of hACE2-transduced BALB/c mice therapeutically treated with DZIF-10c. (**A**) Study plan for the therapeutic dose regimen. BALB/c mice were transduced with Ad_ACE2-mCherry three days before the challenge with SARS-CoV-2. Mice were treated i.n. or i.p. with 40 mg/KG body weight DZIF-10c or an IgG control antibody on days one and three post infection. On day four post infection, the animals were euthanized, and samples were collected. (**B**) Infectious SARS-CoV-2 titer in lung homogenates on day four post infection determined by the TCID_50_ assay. (**C**,**D**) SARS-CoV-2 gRNA and sgRNA in lung homogenates on day four post infection determined by RT-qPCR. Error bars represent mean ± SEM. Statistical analyses were performed using Graph Pad Prism and the Mann–Whitney test. *: *p* ≤ 0.05; **: *p* ≤ 0.01. Dotted lines indicate the lower limit of detection. (**E**) Histopathological analysis of the lungs by H&E staining and in situ hybridization of viral RNA. Images were acquired at a magnification of 40× or 100×. (**F**,**G**) Quantification of histopathological scores for dissemination and highest severity of inflammation and dissemination of viral RNA. Error bars represent mean ± SEM. Statistical analyses were performed using Graph Pad Prism and the Mann–Whitney test. No statistical difference was observed in any group.

To investigate the efficiency of a therapeutic treatment with DZIF-10c, we transduced BALB/c mice intratracheally with Ad_ACE2-mCherry three days before the animals were infected intranasally with SARS-CoV-2 (Figure 5A). In the therapeutic regimen, the mice received two doses of 40 mg/KG body weight of DZIF-10c or the isotype control antibody on days one and three after infection, either via the i.p. or the i.n. route. On day four, the animals were sacrificed, and samples were taken. Clinical symptoms were absent except in one mouse of the i.n. control group, which lost 7% body weight on day four (Figure S8). On average, 9.31 × 10^3^ (±3.37 × 10^3^) TCID_50_/25 mg tissue were detected in the i.n. control group and 1.66 × 10^4^ (±4.90 × 10^3^) TCID_50_/25 mg tissue in the i.p. control group (Figure 5B). Similar to the prophylactic regimen, no infectious particles were detected in lung homogenates of DZIF-10c-treated animals indicating efficient neutralization of SARS-CoV-2 after therapeutic intervention with DZIF-10c. Interestingly, SARS-CoV-2 gRNA and sgRNA were only slightly reduced (2-3-fold) in animals receiving DZIF-10c compared to those receiving the isotype control (Figure 5B,C). This observation was confirmed by in situ hybridization, which revealed a slight reduction of viral RNA in lung slices, independent of the route of antibody application (Figure 5E,G). H&E staining revealed widespread inflammation in multiple, partially confluent foci with infiltration of lymphocytes and macrophages (Figure 5E,F). The histopathological picture did not significantly differ between the DZIF-10c-treated and the control groups with a tendency towards a slight improvement after DZIF-10c treatment.

Taken together, therapeutic treatment of SARS-CoV-2 infected hACE2-transduced mice with DZIF-10c, either administered i.p. or i.n., led to a complete neutralization of infectious SARS-CoV-2 and slightly reduced amounts of viral RNA in the lungs of treated animals.

### 3.5. DZIF-10c Shows No Evidence of Enhanced Infection in an In Vitro ADE Assay

Based on observations primarily made with animal models infected with SARS-CoV [39,50], it was discussed whether ADE might play a role during SARS-CoV-2 infection. To address this question, we infected CD14^+^ human blood-derived macrophages with SARS-CoV-2 in the presence of DZIF-10c at neutralizing or non-neutralizing concentrations. To control for baseline infection and general susceptibility of our primary cell culture system, an isotype control antibody and infection with MERS-CoV, which is known to infect human macrophages, were included [51,52]. In addition, VeroE6 cells were included as a permissive cell line to confirm that the selected antibody concentrations were suitable to either neutralize or not neutralize the input virus. The detection of infectious MERS-CoV and high levels of MERS-CoV genome copies in the supernatants of both VeroE6 cells and CD14^+^ human macrophages confirmed that both cell types were susceptible to emerging coronaviruses (Figure S10). As expected, neutralizing concentrations of DZIF-10c efficiently impaired the infection of VeroE6 cells with SARS-CoV-2. After infection with SARS-CoV-2, significantly less viral genome copies were detected in CD14^+^ human macrophages compared to VeroE6 cells (Figure S10A) and no infectious virus could be isolated from the supernatants at either of the tested conditions (Figure S10B) indicating an abortive infection of the macrophages as it has been reported by others [53,54,55]. Overall, these observations do not indicate relevant FcR-mediated enhancement of SARS-CoV-2 infection caused by DZIF-10c in vitro.

## 4. Discussion

Neutralizing antibodies can be effective tools for prevention and treatment of COVID-19 [24]. Our previous work identified a large panel of human monoclonal S-binding antibodies that were isolated from memory B cells from twelve convalescent COVID-19 patients [25]. In the present study, we characterized one antibody of this panel (DZIF-10c) with respect to its S-binding and SARS-CoV-2-neutralizing capacity and assessed the prophylactic and therapeutic efficacy in hACE2-transduced mice. In addition, we compared systemic and topical application of DZIF-10c to investigate alternative routes of administration.

Generally, treatment efficacy of nAbs is dependent on several factors such as the neutralizing titer, antibody half-life and the route of delivery [24]. Our results show that DZIF-10c exerts binding and neutralization potencies comparable to antibodies that showed efficacy in clinical trials [18,19,20]. Moreover, we analyzed the pharmacokinetics of DZIF-10c in NRG and huFcRn mice, two transgenic animal models that previously allowed the reliable prediction of pharmacokinetic profiles of antiviral mAbs in humans [6,7,41,42,43]. When compared to two anti-HIV-1 antibodies (3BNC117 and 10-1074), which displayed half-lives of 2–3 weeks in humans [7,42], DZIF-10c showed similar or even prolonged stability. Exceptionally high neutralizing titers combined with a favorable pharmacokinetic profile therefore suggest that DZIF-10c is suitable for clinical use.

Mechanistically, structural analysis of the DZIF-10c Fab/RBD complex by cryo-EM revealed that DZIF-10c does not directly target the ACE2 binding motif like the majority of neutralizing antibodies but a closely adjacent region in the prefusion conformation of SARS-CoV-2 S. Due to the broad variance of resolution as evident from the local resolution map (Figure S4C), the reconstruction in the binding region (6–10 Å) did not allow for atomic or per amino acid residue refinement of the coordinates. Therefore, the presented model represents only an approximated binding position and angle of the Fab fragment on the RBD. However, the complete loss of activity against pseudoviruses bearing the K444Q mutation indicates that the epitope is probably located in close proximity to this position. One possibility is that K444 is located directly within the epitope and forms a key contact to the complementarity-determining region (CDR) of DZIF-10c, which is disrupted in the case of Q444. On the other hand, it is also conceivable that K444 is found outside of the epitope but a substitution with Q leads to a conformational change in the RBD, thereby interfering with DZIF-10c attachment in an indirect manner. Both models would be in line with the observed binding of DZIF-10c adjacent to the RBM, which encompasses amino acids 437-508. In a recent publication, Barnes et al. proposed a classification scheme for SARS-CoV-2-RBD-specific antibodies based on binding positions and structural properties [13]. Regarding its binding position, DZIF-10c belongs to class 3 binders, which are characterized by targeting a region outside of the ACE2 binding motif (Figure S5). However, in contrast to the other binders in class 3, DZIF-10c is attached to the RBD only in the “up”-conformation, which fits to an alternative classification scheme in which these “up only” binders comprise a subcategory of “RBD core”-specific antibodies [56]. The underlying mechanisms of neutralization can differ between these antibodies, including a destabilization of the pre-fusion S [57,58] and a steric clashing with hACE2 [59,60]. It remains possible that also further, hitherto unknown mechanisms play a role, e.g., blocking the conformational changes, which are needed for the initiation of membrane fusion. Further studies are necessary to define both the precise epitope with atomic details and the exact mode of action of DZIF-10c.

In order to show the in vivo efficacy of DZIF-10c, we demonstrated that both therapeutic and prophylactic application in SARS-CoV-2-infected hACE2-transduced BALB/c mice fully neutralized the infectious virus in the mouse lungs. In the prophylactic setting, the magnitude of viral load reduction and the reduced histopathology are in line with previous reports of other mouse transduction models [22,47]. Consistent with earlier reports from nAb-treated or vaccinated animals [17,61], we could further observe a dramatic decrease of sgRNA compared to control mice. Since the remaining gRNA levels could potentially originate from input virus or neutralized particles, sgRNA only appears during active replication [62,63] indicating that DZIF-10c protected these animals from SARS-CoV-2 infection. After therapeutic treatment with DZIF-10c, we observed less differences in viral genome copies even though the infectious virus was completely neutralized. This may be explained by the transduction mouse model that only partially reflects human SARS-CoV-2 infection [22,64,65]. Since only successfully transduced cells are susceptible, virus spread is abolished as soon as all transduced cells have been infected. If the whole susceptible cell pool would have already been infected on day 1, the therapeutic intervention with DZIF-10c would have come too late to see a difference in virus spread. This might be the reason that we only observed an efficient neutralization of progeny infectious virus, but less difference in the qPCR and in histology. Furthermore, this potential early stop in virus spread may also explain why even untreated control mice challenged with SARS-CoV-2 did not develop overt clinical symptoms. Nevertheless, hACE2-transduced mice consistently display productive infection with SARS-CoV-2 indicated by high levels of viral gRNA and sgRNA, infectious progeny virus and interstitial pneumonia and thus represent an important and useful animal model for SARS-CoV-2. Another explanation for the only minor differences regarding lung pathology in the therapeutic setting could be that the end point on day 4 was chosen too early. Maybe, a later end point, e.g., day 7, would have allowed the inflammation to spread more broadly in the lungs of control mice so that the therapeutic effect of DZIF-10c would have been clearer. Future studies should further include the analysis of inflammation markers or cytokines in serum or BAL.

One significant advantage of nAb immunotherapies over vaccination is their ability to provide immediate protection. However, the route of delivery can have a substantial impact on the bioavailability and the clinical efficacy of a nAb. This is especially true for respiratory viruses that are predominantly present in the lung lumen. While systemic administration of nAbs leads to high antibody concentrations in serum, the availability in the lung lumen is much less pronounced, thus entailing the risk that the antibody levels at the site of action are below the effective dose [24]. Several studies reported superiority of intranasal nAb delivery over systemic application in RSV or influenza A virus animal models [66,67,68], which is in line with a very recent study in hamsters showing the efficacy of an inhaled neutralizing antibody against SARS-CoV-2 [69]. Our results indicate that prophylactic treatment with DZIF-10c conveys protection against infection with SARS-CoV-2 after systemic and topical application. While no infectious particles could be detected in either setting, the magnitude of decrease of viral gRNA and sgRNA and the improvement in lung pathology were more pronounced in animals after i.n. treatment. In our study, the administration of DZIF-10c via the i.n. route was supposed to mimic a topical delivery into the respiratory tract as the primary site of infection. During i.n. administration, the volume of the fluid and the state of consciousness of the animals can have a substantial impact on the distribution of the applied substance. For instance, alert mice would ingest the majority of i.n. administered substances leading to an unintended delivery to the gastrointestinal tract. In contrast, the administered fluid stays in the nasal cavity or reaches the respiratory tract in anesthetized mice, depending on the volume [70]. Although we did not analyze the exact biodistribution of DZIF-10c in i.n. treated mice, other studies have shown that i.n. application of approximately 30 µL into the nostrils of anesthetized mice leads to a reliable delivery of monoclonal antibodies into the upper and lower respiratory tract [70,71]. Intranasal application of antibodies has previously been shown to result in FcRn-mediated uptake and permeation of IgG through the nasal airway mucosa [72,73]. This can potentially lead to partial degradation of applied antibodies reducing the bioavailability in the lung. On the other hand, transmucosal uptake of antibody–antigen complexes may also have the potential to trigger processing by antigen-presenting cells followed by the induction of an immune response in local lymphoid follicles [72,74]. It cannot be excluded that this process has had an additional beneficial impact on DZIF-10c treated mice in our study. These aspects of intranasal delivery in our preclinical model therefore have to be taken into account when translating the results into a clinical study using oral inhalation of aerosolized antibodies. Overall, our results indicate that DZIF-10c shows solid antiviral efficacy against SARS-CoV-2 when administered topically.

The recent emergence of SARS-CoV-2 variants with enhanced transmissibility and mutations in the RBD that potentially lead to immune escape raises concerns regarding the effectiveness of vaccines and nAbs. In particular, the single point mutations K417N, E484K and N501Y have been shown to completely abolish the neutralizing activity of several monoclonal antibodies [35,36,37,75]. Furthermore, a recent study demonstrated that two nAbs already in clinical use completely lost their activity against VOC B.1.351 that harbors all three of the above-mentioned substitutions [37,38]. Due to the pivotal importance of viral escape, we assessed the impact of these mutations on the neutralizing capacity of DZIF-10c. Using a pseudovirus assay, we showed that the neutralization potency of DZIF-10c was not affected by 16 out of 19 tested point mutations in the RBD, including the K417E and N501Y mutation, and DZIF-10c retained reduced activity against pseudoviruses harboring the E484K mutation. The only mutation that led to a complete loss of activity in the pseudovirus assay was the K444Q mutation. This substitution is not present in circulating VOCs but was specifically selected when recombinant vesicular stomatitis virus carrying SARS-CoV-2 S was propagated in the presence of monoclonal antibodies or convalescent plasma [17,76]. Thus, K444Q might display a potential escape mutation and should be thoroughly monitored during further development of DZIF-10c. Importantly, however, experiments with authentic viruses of VOCs demonstrated that DZIF-10c remained fully active against VOC B.1.1.7 and retained activity against VOC B.1.351, although with approximately 17-fold reduced potency.

When it comes to immunotherapy against viral pathogens, another area of concern is antibody-dependent enhancement of infection and disease, which is based on the uptake of antibody-bound viral particles into FcR-expressing cells like macrophages or dendritic cells [50,77]. To date, there is no clear evidence that ADE plays a significant role in COVID-19 disease progression. Consistent with other studies [21,78], we did not observe any signs of enhanced disease after DZIF-10c treatment in our mouse model (Figures S7 and S8). Furthermore, an in vitro ADE assay did not indicate that DZIF-10c leads to enhanced infection in FcR-bearing human macrophages. Although susceptibility of CD14^+^ human macrophages to SARS-CoV-2 may be limited, this suggests that ADE-related adverse effects of DZIF-10c are not very likely to appear in clinical trials in humans.

In summary, we characterized a novel fully human SARS-CoV-2 neutralizing antibody, DZIF-10c, which exhibited an extraordinary neutralizing potency comparable with some of the most potent anti-SARS-CoV-2 nAbs available to date. We further demonstrated that prophylactic treatment with DZIF-10c protects hACE2-transduced mice from infection with SARS-CoV-2. Moreover, our data indicate that topical administration of DZIF-10c may be a suitable delivery route that could be advantageous in clinical use. The results presented in this study made a decisive contribution to DZIF-10c having entered a phase I/II clinical trial that, for the first time, investigated an inhaled administration of a nAb targeting SARS-CoV-2 (NCT04631705).

## 5. Patents

A patent application encompassing DZIF-10c was filed by the University of Cologne, listing F.K., S.B., C.K., M.Z. and H.G. as inventors (20182325.9).

## Data Availability

All relevant data are within the manuscript and its Supporting Information files. Materials/samples used in the analysis described in this manuscript may be made available to qualified, academic, noncommercial researchers through a material transfer agreement upon request.

## Supplementary Materials

The following are available online at https://www.mdpi.com/article/10.3390/v13081498/s1. Supplementary Methods [79,80,81,82,83,84,85,86,87]. Figure S1: Binding of DZIF-10c to different forms of SARS-CoV-2 S; Figure S2: Affinity analysis of DZIF-10c and SARS-CoV-2 S RBD-HIS measured by SPR; Figure S3: Neutralization of SARS-CoV-2 pseudoviruses bearing different mutations in the spike RBD; Figure S4: Resolution of the cryo EM reconstruction; Figure S5: Classification scheme for SARS CoV 2 RBD specific antibodies according to Barnes et al; Figure S6: RT-qPCR-based analysis of mCherry levels in lung homogenates of hACE2-transduced mice; Figure S7: Body weight and clinical scores of prophylactically treated SARS-CoV-2 infected mice; Figure S8: Body weight and clinical Scores of therapeutically treated SARS-CoV-2 infected mice; Figure S9: Representative examples for histopathology scoring; Figure S10: In vitro ADE assay.
