# Supplementary material for: Intranasal Administration of a Monoclonal Neutralizing Antibody Protects Mice against SARS-CoV-2 Infection
Source: Viruses. 2021 Jul 29;13(8):1498. doi: 10.3390/v13081498 (PMC8402634; doi:10.3390/v13081498)
Supplement: Supplementary file 1 [file viruses-13-01498-s001.zip › Halwe_Suppl. Figures.pdf]

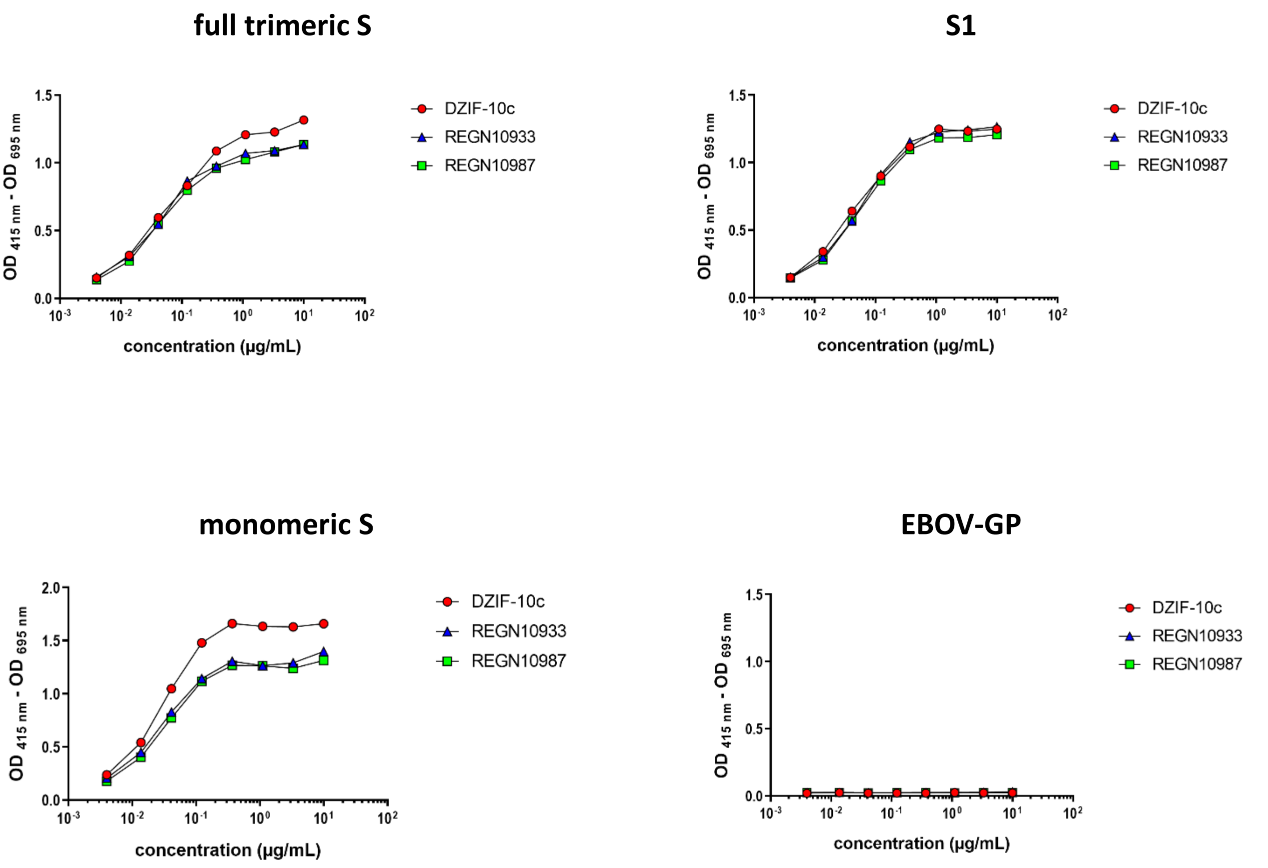

Fig. S1: Binding of DZIF-10c to different forms of SARS-CoV-2 S

Interaction of DZIF-10c with full trimeric S **(A)**, S1 **(B)**, monomeric S **(C)** and EBOV-GP **(D)** measured by ELISA.

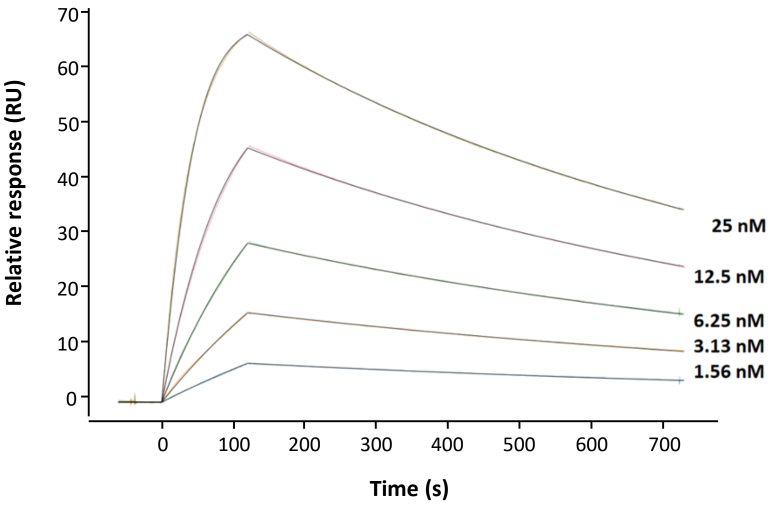

Fig. S2: Affinity analysis of DZIF-10c and SARS-CoV-2 S RBD-HIS measured by SPR.

DZIF-10c (1 µg/mL) was captured on the Protein A/G surface and the analyte was injected over the captured ligand. The concentrations of the analyte (SARS-COV-2 RBD-His) were as follows: 0 nM, 1.56 nM, 3.13 nM, 6.25 nM, 12.5 nM, and 25 nM. The analyte interaction with sensor surface (flow cell 1) and blank (HBS-EP+ or 0 nM analyte) were subtracted from the raw data. Sensorgrams were then fit globally to 1:1 Langmuir binding to provide on-rate (ka), off-rate (kd), and affinity (KD) values. The diagram shows a representative SPR sensorgram from three experiments.

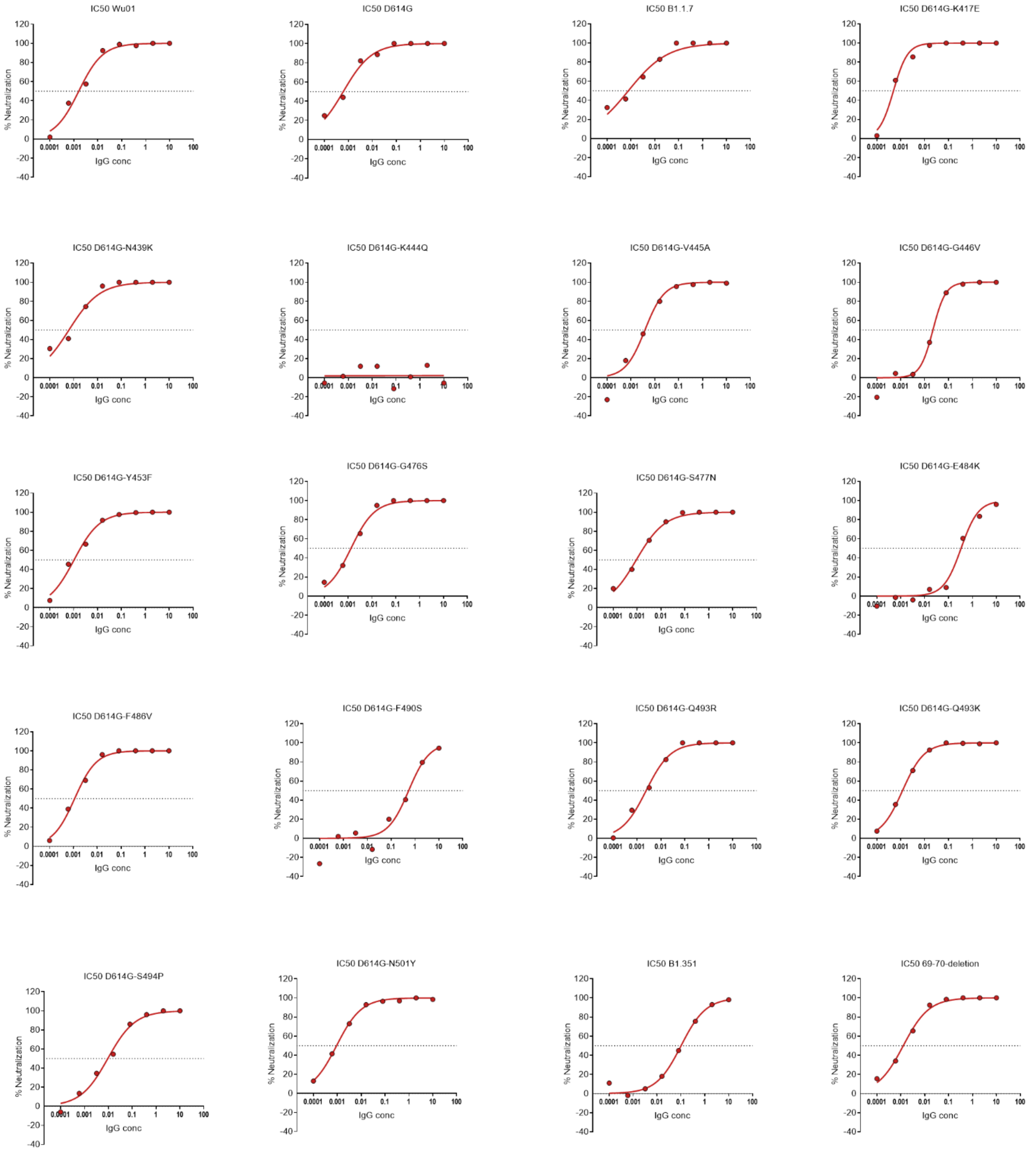

Fig. S3: Neutralization of SARS-CoV-2 pseudoviruses bearing different mutations in the spike RBD

Neutralizing activity of DZIF-10c against SARS-CoV-2 pseudoviruses bearing S proteins with point mutations found in circulating variants of concern. The dotted line indicates 50% neutralization (IC_{50}).

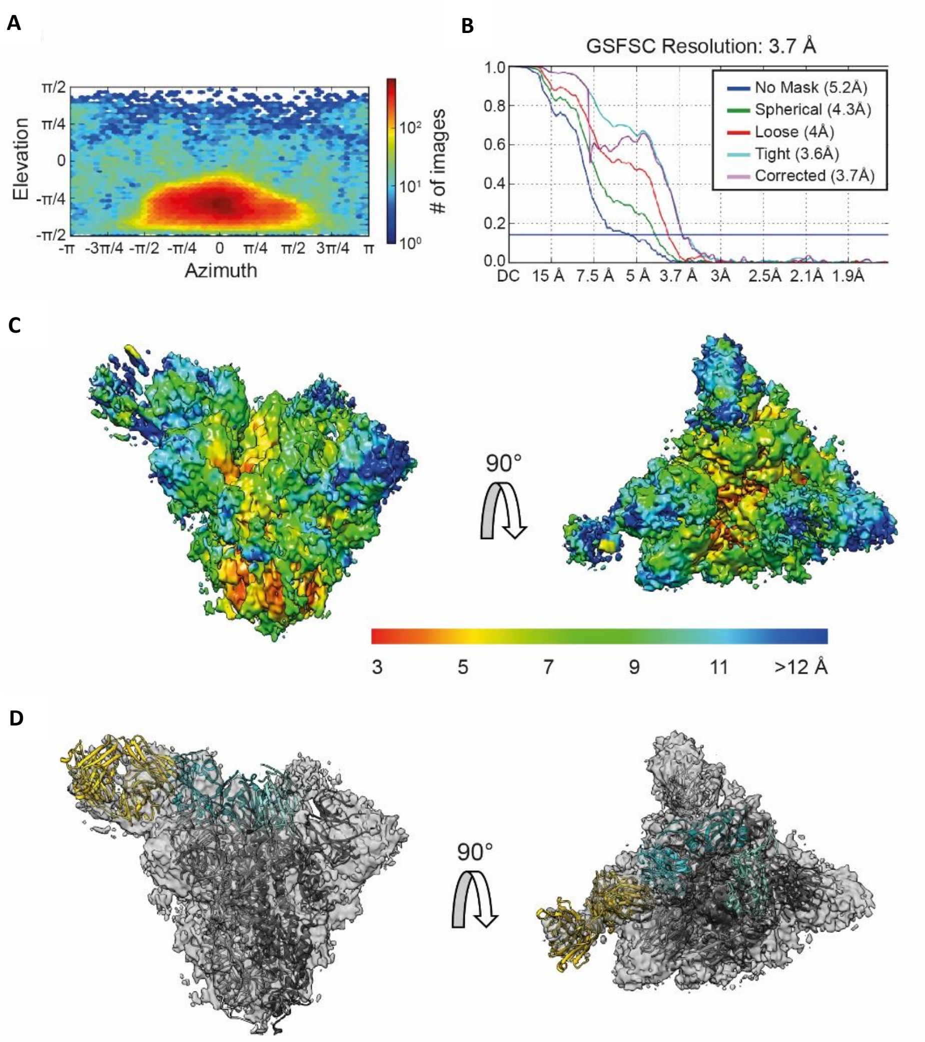

Figure S4: Resolution of the Cryo-EM reconstruction.

(A) Viewing Direction Distribution heat map, showing the predominance of top views. **(B)** Gold standard FSC curve. The 0.143 criterion reports a global resolution of 3.7 Å. **(C)** Local resolution map showing the variance of resolution in the reconstruction of the complex using a rainbow color code from red (high resolution) to blue (low resolution). Left: side view; Right: top view. **(D)** Rigid body fits of PDB models 6VSB (S protein protomers: grey shades; RBDs: cyan shades) and 7C01 (Fab fragment, yellow) overlaid with the reconstructed, transparent cryo-EM map (grey). Left: side view; Right: top view.

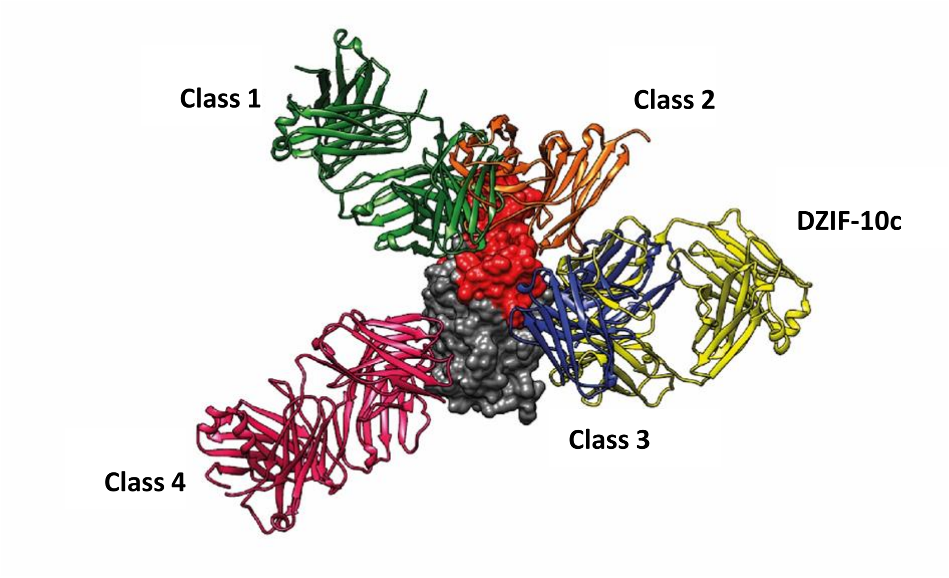

Figure S5: Classification scheme for SARS-CoV-2-RBD-specific antibodies according to Barnes et al. [13]. Based on the binding position, DZIF-10 belongs to class 3 binders. Grey: Spike RBD; Red: ACE-2 binding motif.

A

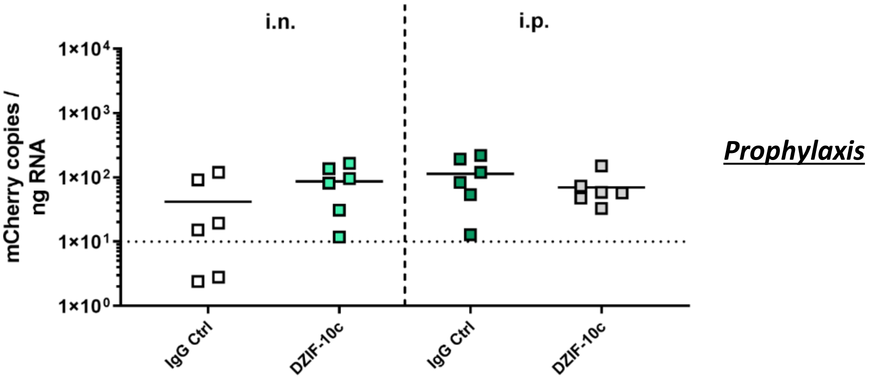

B

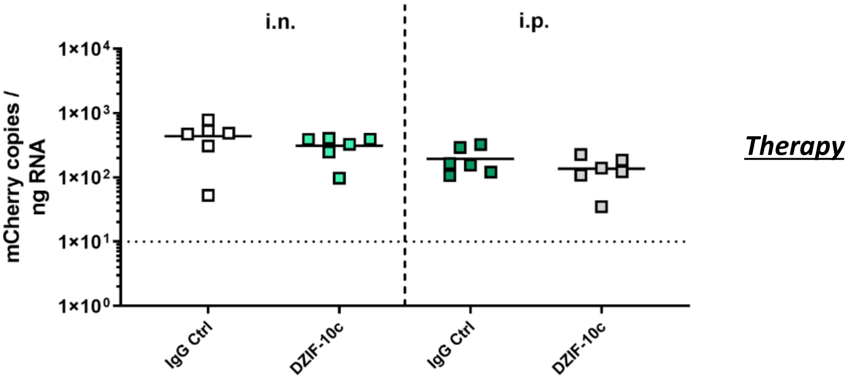

Fig. S6: RT-qPCR-based analysis of mCherry levels in lung homogenates of hACE2-transduced mice

BALB/c mice were transduced with AdV-hACE2 encoding for the reporter mCherry three days before infection. Mice were treated i.n. or i.p. with 40 mg/KG body weight DZIF-10c or an IgG control antibody on day one before **(A)** or on day one and three after **(B)** challenge with SARS-CoV-2. On day four post infection, the animals were euthanized and samples were collected. Squares indicate mCherry mRNA copies in lung homogenates on day four post infection determined by RT-qPCR. The dotted lines show the limit for inclusion at 10 mCherry copies / ng RNA. Two mice from the i.n. control group in the prophylactic study (A) had to be excluded from the analysis due to insufficient transduction efficiency indicated by mCherry copy numbers below 10 copies / ng RNA.

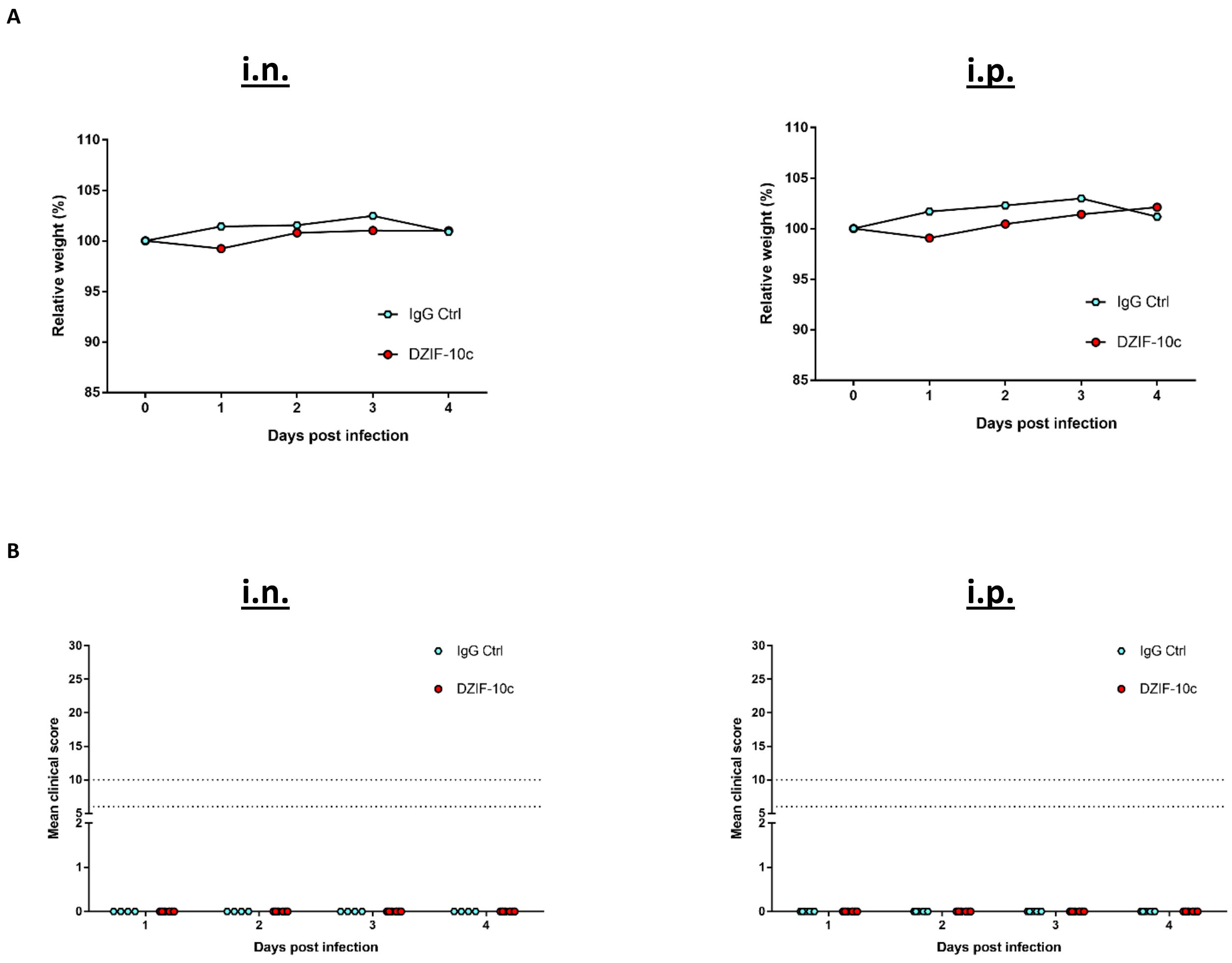

Fig. S7: Body Weight and Clinical Scores of prophylactically treated SARS-CoV-2 infected mice

(A) Changes in body weight for i.n. or i.p. treated mice after SARS-CoV-2 challenge. Dots depict group means at the respective time point. **(B)** Clinical scores for i.n. or i.p. treated mice after SARS-CoV-2 challenge. Clinical scores are composed of body weight, spontaneous behavior and general condition. Dotted lines represent clinical end points. Animals are euthanized at a score of 10 or of 6 on two consecutive days. Dots depict individual scores for each animal at the respective time point.

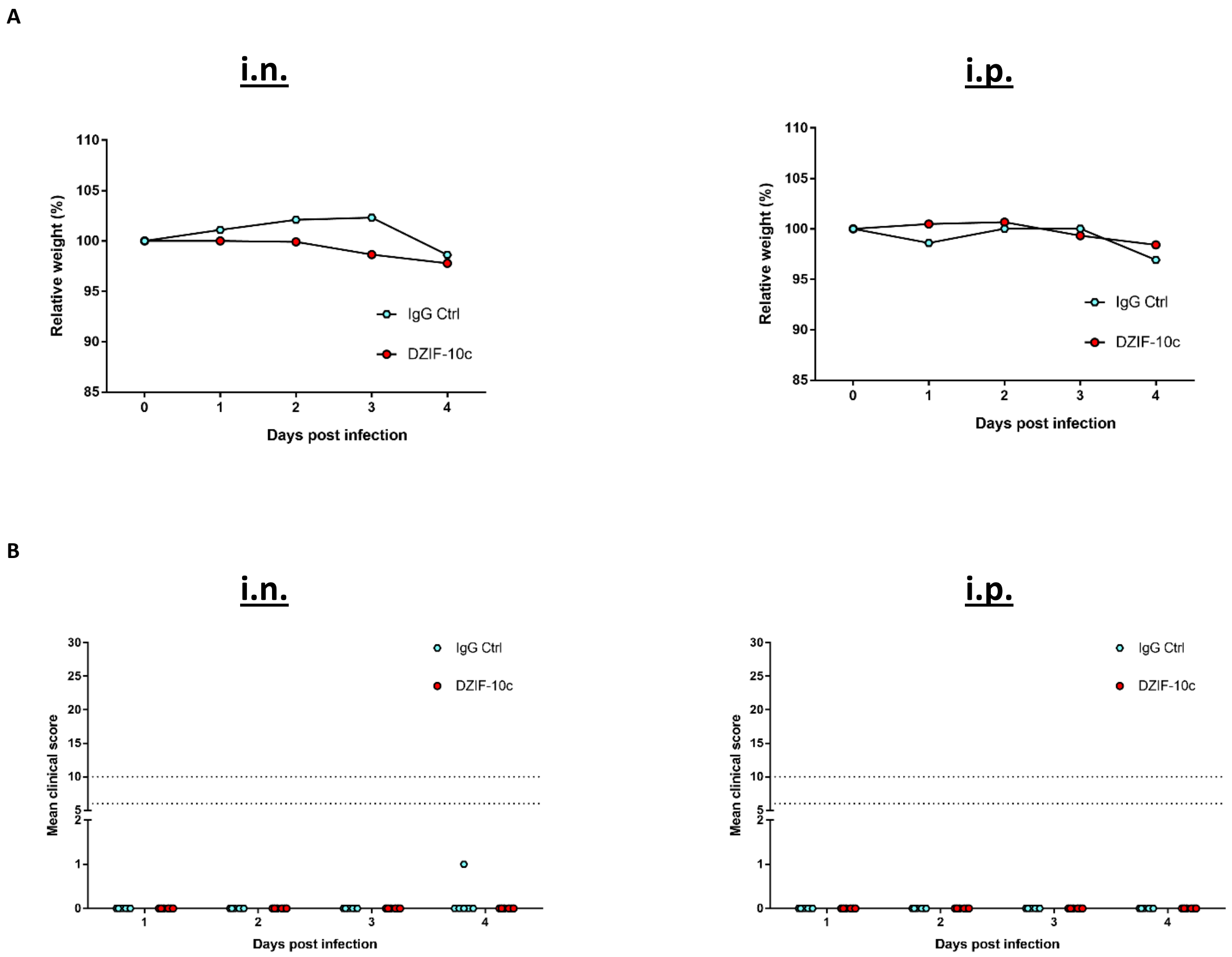

Fig. S8: Body Weight and Clinical Scores of therapeutically treated SARS-CoV-2 infected mice

(A) Changes in body weight for i.n. or i.p. treated mice after SARS-CoV-2 challenge. Dots depict group means at the respective time point. **(B)** Clinical scores for i.n. or i.p. treated mice after SARS-CoV-2 challenge. Clinical scores are composed of body weight, spontaneous behavior and general condition. Dotted lines represent clinical end points. Animals are euthanized at a score of 10 or of 6 on two consecutive days. Dots depict individual scores for each animal at the respective time point.

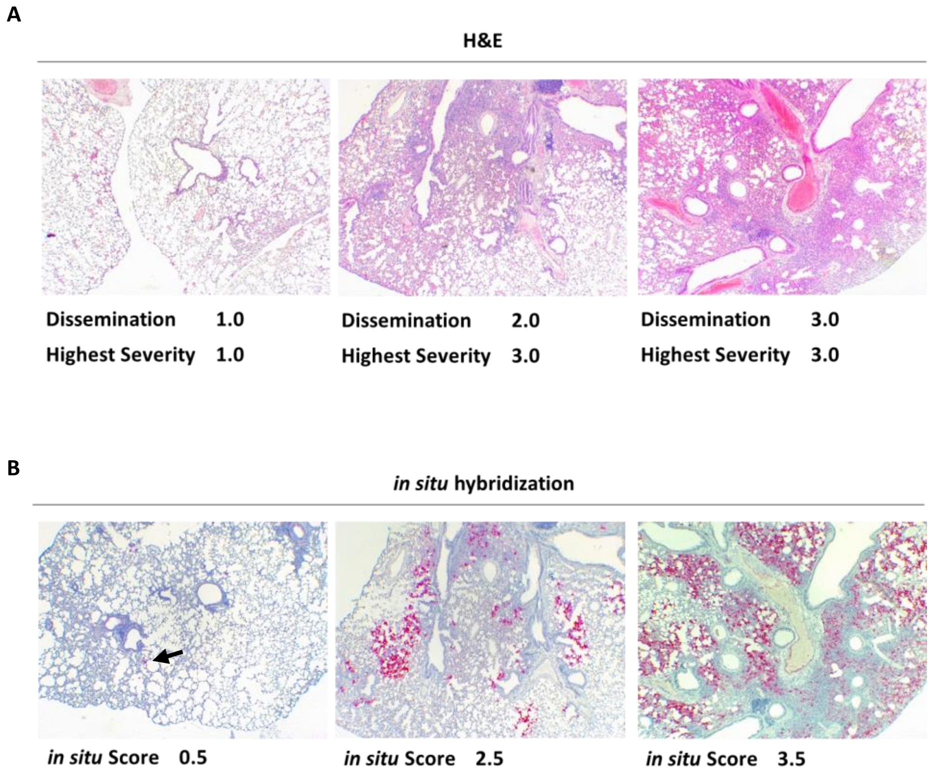

Fig. S9: Representative examples for histopathology scoring

Histopathological analysis of the lungs by H&E staining **(A)** and *in situ* hybridization of viral RNA **(B)**. Images were acquired at a magnification of 40x. Histopathological scores of the respective samples are specified below. The black arrow in Panel B highlights one single spot where viral RNA was detected.

A

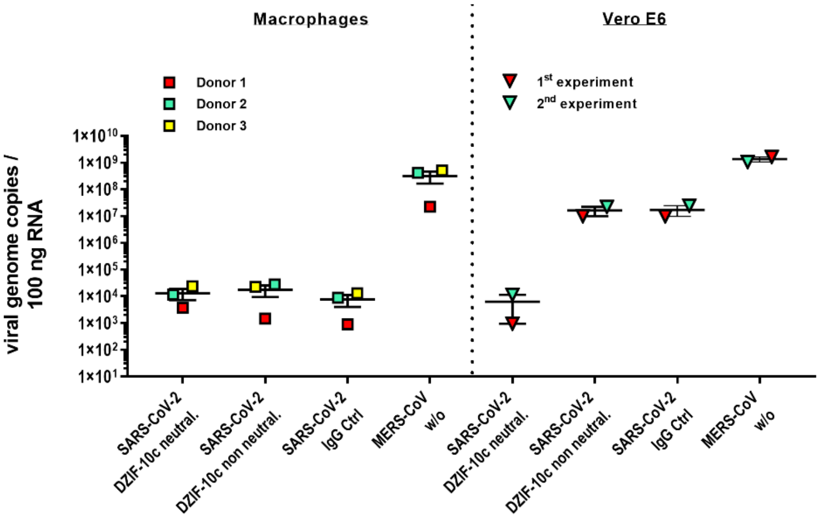

B

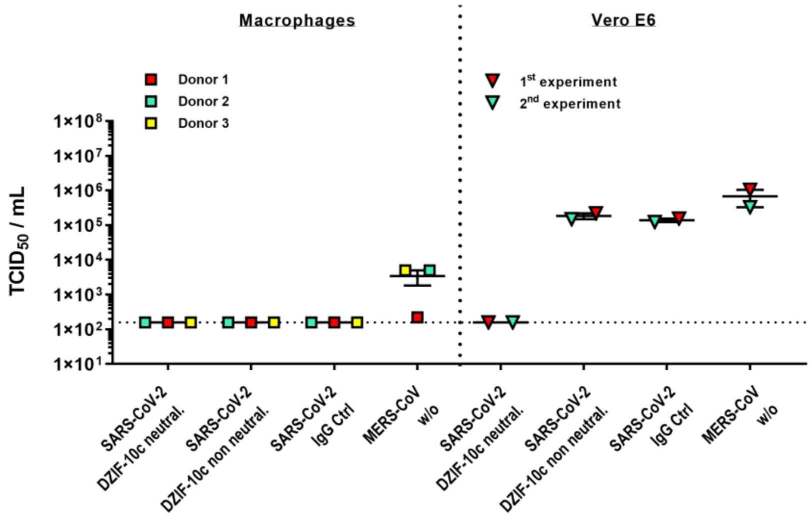

Fig. S10: *in vitro* ADE Assay

$CD14^+$-differentiated human blood macrophages were infected with SARS-CoV-2 in the presence of DZIF-10c at neutralizing and non-neutralizing concentrations or an IgG isotype antibody. General infectivity of macrophages was confirmed by infection with MERS-CoV. VeroE6 cells were included to confirm the adequacy of the selected antibody concentrations and viral infectivity. **(A)** SARS-CoV-2 and MERS-CoV genome copies in cell lysates determined by qRT-PCR. **(B)** Infectious SARS-CoV-2 and MERS-CoV titers in cell culture supernatants determined by TCID50 assay. Error bars represent mean ± SEM. Dotted lines indicate lower limit of detection.
