# Supplementary material for: Intranasal Administration of a Monoclonal Neutralizing Antibody Protects Mice against SARS-CoV-2 Infection
Source: Viruses. 2021 Jul 29;13(8):1498. doi: 10.3390/v13081498 (PMC8402634; doi:10.3390/v13081498)
Supplement: Supplementary file 1 [file viruses-13-01498-s001.zip › Halwe_Suppl. Methods.pdf]

Supplementary Methods

Cell culture

Vero E6 cells (Vero C1008, ATCC, Cat#CRL-1586, RRID: CVCL_0574) were maintained in Dulbecco`s Modified Eagle`s Medium (DMEM) supplemented with 10% FBS, 1% Glutamine and 1% penicillin/streptomycin (Gibco). Virus infections were performed in DMEM with 2% FBS, 1% Glutamine and 1% penicillin/streptomycin.

Cultivation and differentiation of human blood-derived macrophages was performed at the Institute for Lung Research (Marburg, Germany) as described previously [79,80]. Briefly, macrophages were obtained from primary monocytes isolated by MACS CD14 positive selection from healthy donor buffy coats provided by the Centre for Transfusion Medicine and Haemotherapy, University Hospital Giessen and Marburg (Germany). All blood donors gave informed written consent for use of their blood samples for scientific purposes (Ethics approval number: 161/17). Freshly isolated monocytes were seeded in ultra-low attachment plates (Corning) and were left to adhere for 2 h in supplement-free RPMI medium (Gibco). After adhesion of cells, differentiation was initiated by addition of 1% human AB serum (Sigma) and monocytes were cultivated for seven days. Maturation to blood-derived macrophages was confirmed by microscopy. Cells were detached by incubation with pre-warmed PBS for 10 min and then seeded at a density of $3x10^5$ cells/12well in 1 mL of fresh RPMI media. All cells were cultivated at 37°C and 5% CO_2.

Viruses

All work with infectious SARS-CoV-2 was performed in the BSL-4 facility at the Institute of Virology, Philipps University Marburg (Marburg, Germany). BavPat1/2020 isolate (#026V-03883) was purchased from European Virus Archive Global (EVAg). The B.1.1.7 variant (BioProject no. PRJNA721582) was a clinical isolate and was isolated at the Institute of Virology, Philipps University Marburg (Marburg, Germany). The B.1.351 (GenBank Accession no. MW822592) variant was a clinical isolate and was isolated at the Institute for Medical Virology, University Hospital Frankfurt (Goethe University Frankfurt am Main, Germany). All

viruses were propagated on Vero76-TMPRSS2 cells using DMEM supplemented with 2% FBS, 1% Glutamine and 1% penicillin/streptomycin (Gibco). On day two (BavPat1/B.1.1.7/B.1351) after infection, cell culture supernatants were harvested, and titers were determined by immunoplaque and $TCID_{50}$ assay on VeroE6 cells.

Surface Plasmon Resonance (SPR)

The SPR assays were performed using a Biacore 8K system and a CM5 sensor chip (GE Healthcare). The running buffer for this experiment and all dilutions were done in 1 X HBS-EP+ (GE Healthcare Life Sciences BR100669). The CM5 sensor chip was activated with equal mixture of EDC/NHS for 420 sec at a flow rate of 10 ml/min and immobilized with Protein A/G (50 mg/ml in 10 mM acetate pH 4.5) for 420 sec at a flowrate of 10 ml/min resulting in ≈ 2400-2800 RU of Protein A/G on the surface. Subsequently, the sensor chip was deactivated with 1M ethanolamine HCl for 420 sec at a flowrate of 10 ml/min. DZIF-10c (1 mg/ml) was captured on the Protein A/G surface for 60 sec at a flowrate of 10 ml/min resulting in capture levels of ≈ 150 RU. The analyte (SARS-COV2 RBD-His) was injected over the captured ligand for 120 sec at a flowrate of 30 ml/min. The dissociation was done for 600 sec. The concentrations of the analyte were as follows: 0 nM, 1.56 nM, 3.13 nM, 6.25 nM, 12.5 nM, and 25 nM. After each analyte injection was complete, the surface was regenerated by injecting 0.85% phosphoric acid for 30 sec at a flowrate of 30 ml/min. The analyte interaction with sensor surface (flow cell 1) and blank (HBS-EP+ or 0 nM analyte) were subtracted from the raw data. Sensorgrams were then fit globally to 1:1 Langmuir binding to provide on-rate (k_a), off-rate (k_d), and affinity (K_D) values. The binding experiments for SARS-COV-2 RBD-His were performed three separate times using fresh dilutions.

ELISA analysis

ELISA plates were coated with 2 mg/ml of SARS-CoV-2 spike ectodomain, RBD, N-terminal truncated, or EBOV Makona glycoprotein (GP) ectodomain in PBS or monomeric SARS-CoV-2 spike ectodomain in 2 M Urea at 4°C overnight. Proteins were produced as previously described [25]. Next day, plates were blocked

with 5% BSA in PBS for 60 min at RT, incubated with primary antibody (starting concentration 10 μg/ml) in PBS for 120 min and secondary antibody (anti-human IgG-HRP; Southern Biotech 2040-05) diluted 1:2500 in 1% BSA in PBS for 60 min at RT. ELISAs were developed with ABTS solution (Thermo Fisher 002024) and absorbance was measured at 415 nm and 695 nm.

Pseudovirus neutralization assay

SARS-CoV-2 pseudovirus particles were generated by co-transfection of individual plasmids encoding HIV Tat, HIV Gag/Pol, HIV Rev, luciferase followed by an IRES and ZsGreen, and the SARS-CoV-2 spike protein [33] into HEK 293T cells using the FuGENE 6 Transfection Reagent (Promega). Spike sequences from the following global strains REF were used: Wu01 spike (EPI_ISL_40671); BavP1 spike (EPI_ISL_406862); ARA36 spike (EPI_ISL_418432); DRC94 spike (EPI_ISL_417947); CA5 spike (EPI_ISL_408010) and NRW8 spike (EPI_ISL_414508), B.1.1.7 variant [81] and B.1.351 variant [82]. Mutations were introduced by PCR into the Wu01 spike as backbone. Virus culture supernatant was harvested at 48 h and 72 h post transfection and stored at -80°C till use. The harvested virus was titrated by infecting 293T expressing ACE2 and after a 48 h incubation at 37°C and 5% CO2, luciferase activity was determined after addition of luciferin/lysis buffer (10 mM MgCl2, 0.3 mM ATP, 0.5 mM Coenzyme A, 17 mM IGEPAL (all Sigma-Aldrich), and 1 mM D-Luciferin (GoldBio) in Tris-HCL) using a microplate reader (Berthold). For neutralization assays, a virus dilution with a relative luminescence units (RLU) of approximately 1000-fold in infected cells versus non-infected cells was selected. For testing neutralization potency of DZIF-10c, serial dilutions of DZIF-10c were co-incubated with pseudovirus supernatants for 1 h at 37°C, following which 293T-ACE-2 cells were added. After a 48-hour incubation at 37°C and 5% CO2, luciferase activity was determined using the luciferin/lysis buffer. After subtracting background RLUs of non-infected cells, 50% inhibitory concentration (IC50) were calculated as the DZIF-10c concentration resulting in a 50% reduction in RLU compared to the untreated virus control wells. Each antibody dilution was tested in duplicates. IC50 values were calculated by plotting a dose response curve in GraphPad Prism 7.0.

SARS-CoV-2 Virus neutralization test (VNT100)

SARS-CoV-2 neutralizing activity of human monoclonal antibodies was investigated based on a previously published protocol with slight modifications [25,83,84]. Briefly, monoclonal antibodies were serially diluted in DMEM supplemented with 2% FBS, 1% glutamine and 1% penicillin/streptomycin (Gibco) in 96-well plates before 100 PFU SARS-CoV-2 were added to each sample. Subsequently, Vero E6 cells (Vero C1008, ATCC, Cat#CRL-1586, RRID: CVCL_0574) were washed with PBSdef, trypsinized and diluted in DMEM with 10% FBS, 1% glutamine and 1% penicillin/streptomycin. Cells were diluted in DMEM to a final concentration of 2% FBS, 1% glutamine and 1% pen/strep and 100 µl of the cell solutions was added to virus/antibody samples, corresponding to approximately 20.000 cells/well. Neutralization was defined as absence of cytopathic effect compared to virus controls (IC100). The following controls were included: back titration of virus dilution, positive control as an inter-assay neutralization standard (cells infected with SARS-CoV-2 and treated with an antibody with a known neutralizing titer), negative control (cells without infection and antibodies), cytotoxicity control (cells without infection, only treated with antibodies).

Tissue culture infectious dose 50 (TCID50) Assay

The amount of infectious SARS-CoV-2 particles from cell culture supernatants or lung homogenates was determined by TCID50 assay. Vero E6 cells were cultured in DMEM with 2% FBS, 1% glutamine, 1% penicillin/streptomycin in 96-well plates and were inoculated with 10-fold serial dilutions of supernatant samples or 5-fold serial dilutions of lung homogenates. At four days post infection, SARS-CoV-2 CPE was evaluated, and titers per ml or 25 mg lung tissue were calculated using the Spearman-Kaerber method [85].

Detection of genomic RNA (gRNA) by quantitative real-time reverse transcription PCR (RT-qPCR)

In order to quantify viral gRNA, nucleic acids were isolated from cell lysates or lung homogenates using the RNeasy Mini Kit (Qiagen) according to manufacturer's instructions. Total RNA amount was measured using a NanoDrop ND-100 spectrophotometer.

For analysis of SARS-CoV-2 genome copies, RNA was reverse transcribed and viral genome copies quantified by real-time PCR using the OneStep RT-PCR Kit (Qiagen) and the StepOne Real-Time PCR System (Applied Biosystems). Primers and probes targeting the E gene of SARS-CoV-2 (E Assay_First Line Screening) as well as a positive control plasmid were purchased from idtdna (Berlin, Germany) [86]. Reverse transcription and amplification were performed using the following protocol: 55°C for 30 min, 95°C for 15 min followed by 45 cycles of 95°C for 5 s, 60°C for 15 s and 72°C for 15 s. Quantification was carried out using a standard curve based on 10-fold serial dilutions of a plasmid DNA comprising the target region ranging from 103 to 106 copies.

E_Sarbeco_F1: ACAGGTACGTTAATAGTTAATAGCGT

E_Sarbeco_R2: ATATTGCAGCAGTACGCACACA

E_Sarbeco_P1: FAM-ACACTAGCCATCCTTACTGCGCTTCG-BBQ

MERS-CoV genome copies were determined using a previously published protocol [29,87]. Briefly, RNA was reverse transcribed and viral genome copies quantified by real-time PCR using the SuperScript III OneStep RT-PCR Kit (Invitrogen Life Technologies) and the StepOne Real-Time PCR System (Applied Biosystems). Primers and probes targeting the E gene of MERS-CoV were purchased from Tib-Molbiol (Germany). Reverse transcription and amplification were performed using the following protocol: 55°C for 20 min, 95°C for 3 min followed by 45 cycles of 94°C for 15 s, 58°C for 30 s. Quantification was carried out using a standard curve based on 10-fold serial dilutions of appropriate cloned RNA ranging from 101 to 106 copies.

upE_Fwd: GCAACGCGCGATTCAGTT

upE_Rev: GCCTCTACACGGGACCCATA

upE_Prb: FAM-CTCTTCACATAATCGCCCCGAGCTCG-TAMRA

Detection of subgenomic RNA (sgRNA) by quantitative real-time reverse transcription PCR (RT-qPCR)

Subgenomic RNA was determined according to a previously published protocol [17,62]. Nucleic acids were isolated as described before. SARS-CoV-2 subgenomic RNA of the E gene was reverse transcribed and copy numbers quantified by real-time PCR using the SuperScript III OneStep RT-PCR Kit (Invitrogen Life Technologies) and the StepOne Real-Time PCR System (Applied Biosystems). Primers and probes targeting the leader and the E gene of SARS-CoV-2, respectively, were purchased from Tib-Molbiol (Germany). Reverse transcription and amplification were performed using the following protocol: 55°C for 20 min, 95°C for 3 min followed by 45 cycles of 95°C for 10 s, 56°C for 15 s and 72°C for 15 s. Quantification was carried out using a standard curve based on 10-fold serial dilutions of a plasmid DNA comprising the target region ranging from 101 to 107 copies.

sgLead_SARS2_F: CGATCTCTTGTAGATCTGTTCTC

sgE_SARS2_R: ATATTGCAGCAGTACGCACACA

sgE_SARS2_P: FAM-ACACTAGCCATCCTTACTGCGCTTCG-BBQ

Detection of mCherry mRNA by quantitative real-time reverse transcription PCR (RT-qPCR)

To confirm transduction with AdV-hACE2, mRNA levels of the reporter mCherry was determined according to a previously published protocol [29]. Nucleic acids were isolated from lung homogenates as described before. mCherry mRNA was reverse transcribed and copy numbers quantified by real-time PCR using the SuperScript III OneStep RT-PCR Kit (Invitrogen Life Technologies) and the StepOne Real-Time PCR System (Applied Biosystems). Primers and probes targeting the mCherry gene were purchased from Tib-Molbiol (Germany). Reverse transcription and amplification were performed using the following protocol: 50°C for 30 min, 95°C for 15 min followed by 40 cycles of 95°C for 15 s, 48°C for 30 s and 72°C for 20 s. Quantification was carried out using a standard curve based on 10-fold serial dilutions of appropriate cloned RNA ranging from $5x10^2$ to $5x10^5$ copies.

mcherry181for: CATGGTAACGATGAGTTAG

mcherry287rev: GTTGCCTTCCTAATAAGG

mcherry probe: FAM- TACCACCTTACTTCCACCAATCGG-BBQ

***In vitro* ADE Assay**

For assessment of possible DZIF-10c-related enhancement of SARS-CoV-2 infection (ADE), human blood-derived macrophages were infected with SARS-CoV-2 in presence of different concentrations of DZIF-10c or IgG isotype control antibodies. 2.5×10^5 macrophages were seeded in 12 well plates in 1 ml RPMI medium supplemented with 1% Glutamine, penicillin/streptomycin and non-essential amino acids. After adhesion, 10% FBS was added and the cells were incubated for three to four days. Prior to infection with SARS-CoV-2 non-neutralizing IgG control antibodies or DZIF-10c in neutralizing and sub-neutralizing concentrations, respectively, were incubated for 1 h at 37°C together with 800 50% tissue culture infective doses ($TCID_{50}$) SARS-CoV-2 (BavPat1/2020 isolate, European Virus Archive Global # 026V-03883). Directly before inoculation of macrophages with the antibody/virus mixture, 500 µl of the macrophage cell culture medium was transferred to a new 12 well plate and mixed with fresh supplemented 10% RPMI medium (storage medium). Subsequently, 800 µl of the antibody/virus mixture was added to the macrophages and cells were incubated for 4 h at 37°C. Afterwards, antibody/virus-containing medium was discarded and 1 ml storage medium was added to the cells. At four days post infection, CPE was evaluated and supernatants were collected and stored at -80°C. RNA isolation from cell lysates was performed using the RNeasy Mini Kit (Qiagen) according to manufacturer’s instructions. In control settings, the experimental protocol was modified regarding virus and cells. MERS-CoV (EMC/2012) was used to show general susceptibility of macrophages to infection with coronaviruses. Vero E6 cells were used to demonstrate infectivity of the SARS-CoV-2 isolate as well as the neutralizing activity of DZIF-10c in this experimental setting.
